# The accuracy of artificial intelligence in predicting COVID-19 patient mortality: a systematic review and meta-analysis

**DOI:** 10.1186/s12911-023-02256-7

**Published:** 2023-08-09

**Authors:** Yu Xin, Hongxu Li, Yuxin Zhou, Qing Yang, Wenjing Mu, Han Xiao, Zipeng Zhuo, Hongyu Liu, Hongying Wang, Xutong Qu, Changsong Wang, Haitao Liu, Kaijiang Yu

**Affiliations:** 1 Department of Critical Care Medicine, the First Affiliated Hospital of Harbin Medical University, Harbin, 150001 Heilongjiang Province China; 2 Department of Critical Care Medicine, Harbin Medical University Cancer Hospital, No. 150 Haping Rd, Nangang District, Harbin, 150081 China; 3 Departments of Pharmacy and Cardiology, Harbin Medical University Cancer Hospital, No. 150 Haping Rd, Nangang District, Harbin, 150081 China; 4 Department of Anesthesiology, Harbin Medical University Cancer Hospital, No. 150 Haping Rd, Nangang District, Harbin, 150081 China

**Keywords:** Artificial intelligence, COVID-19, Mortality, meta-analysis

## Abstract

**Background:**

The purpose of this paper was to systematically evaluate the application value of artificial intelligence in predicting mortality among COVID-19 patients.

**Methods:**

The PubMed, Embase, Web of Science, CNKI, Wanfang, China Biomedical Literature, and VIP databases were systematically searched from inception to October 2022 to identify studies that evaluated the predictive effects of artificial intelligence on mortality among COVID-19 patients. The retrieved literature was screened according to the inclusion and exclusion criteria. The quality of the included studies was assessed using the QUADAS-2 tools. Statistical analysis of the included studies was performed using Review Manager 5.3, Stata 16.0, and Meta-DiSc 1.4 statistical software. This meta-analysis was registered in PROSPERO (CRD42022315158).

**Findings:**

Of 2193 studies, 23 studies involving a total of 25 AI models met the inclusion criteria. Among them, 18 studies explicitly mentioned training and test sets, and 5 studies did not explicitly mention grouping. In the training set, the pooled sensitivity was 0.93 [0.87, 0.96], the pooled specificity was 0.94 [0.87, 0.97], and the area under the ROC curve was 0.98 [0.96, 0.99]. In the validation set, the pooled sensitivity was 0.84 [0.78, 0.88], the pooled specificity was 0.89 [0.85, 0.92], and the area under the ROC curve was 0.93 [1.00, 0.00]. In the subgroup analysis, the areas under the summary receiver operating characteristic (SROC) curves of the artificial intelligence models KNN, SVM, ANN, RF and XGBoost were 0.98, 0.98, 0.94, 0.92, and 0.91, respectively. The Deeks funnel plot indicated that there was no significant publication bias in this study (P > 0.05).

**Interpretation:**

Artificial intelligence models have high accuracy in predicting mortality among COVID-19 patients and have high prognostic value. Among them, the KNN, SVM, ANN, RF, XGBoost, and other models have the highest levels of accuracy.

**Supplementary Information:**

The online version contains supplementary material available at 10.1186/s12911-023-02256-7.

## Introduction

The ongoing COVID-19 pandemic poses enormous challenges to global public health, health care systems, and economies. As of December 21, 2022, 649 million people have been diagnosed with COVID-19, and more than 6 million related deaths have occurred worldwide [1]. Although COVID-19 mortality rates have been significantly reduced as vaccination rates have increased and several treatments have been proposed for COVID-19, the progress of the disease has been rapid due to the high complexity of its characteristics. For patients with underlying diseases or those who cannot be treated in a timely manner, the disease tends to progress faster, and the mortality rate is higher [2, 3]. Therefore, effective and accurate outcome predictions and effective and personalized patient management are increasingly important. However, there is still a lack of tools for predicting the risk of death in COVID-19 patients.

Artificial intelligence (AI) is a fusion technology developed based on computer science, cybernetics, information theory, and other disciplines. It can be used in health care applications such as disease diagnosis, prognostic judgement, image analysis, and big data collection. With the rapid development of AI technology, AI algorithms are gradually being applied in various medical fields, such as (1) disease diagnosis, (2) patient morbidity or mortality risk assessment, (3) disease outbreak prediction and surveillance, and (4) health policy and planning [4, 5].

Several studies have shown that AI has high diagnostic value for the early identification of high-risk patients with COVID-19, improving patient prognosis and helping rapid clinical prescreening and triage [6–9]. However, evidence-based medical studies for predicting mortality among COVID-19 patients are currently unavailable. In this study, a meta-analysis on AI prediction of mortality in COVID-19 patients was conducted to guide the early clinical identification of groups with high mortality risk.

## Methods

The present meta-analysis was conducted and reported in accordance with the Preferred Reporting Items for Systematic Reviews and Meta-analyses Statement (PRISMA) guidelines [10]. For further details (Supplementary Material 1 and 2), this meta-analysis has been registered in PROSPERO (CRD42022315158).

### Literature search strategy and screening

The PubMed, Embase, CNKI, Wanfang, China Biomedical Literature Database, VIP, and Web of Science electronic databases were searched from inception to October 2022. Searches were performed by a combination of subject headings and keywords. The search terms included “Artificial Intelligence”, “Machine Intelligence”, “Machine learning”, “AI”, “deep learning”, “random forest”, “Mortality”, “diagnosis”, “SARS-CoV-2”, and “Covid-19”. Two independent researchers (XY and LHX) screened the articles according to the inclusion criteria and performed preliminary screening by reading the titles and abstracts. If a title or abstract could not be judged, the full text was examined to determine whether the article met the inclusion criteria. Disagreements between the researchers were resolved by consulting a third senior expert. For specific retrieval strategies, see Supplementary Material 3.

### Inclusion and exclusion criteria

The inclusion criteria were as follows: (1) the study had to be in English and peer-reviewed; (2) the results of machine learning algorithms and predictions of mortality in COVID-19 patients were provided; (3) the data had to be complete with information on sample size, sensitivity values, and specificity values; (4) the total number of patients with COVID-19 was provided; (5) the study subjects were patients who were confirmed positive for COVID-19 by reverse transcription-polymerase chain reaction (RT–PCR), and there was no age limit; (6) the machine learning models and predictors used in the predictions were clearly described; and (7) a clear overview of the sources of the datasets used in the study was provided.

The exclusion criteria were as follows: (1) documents for which true positive values, false-positive values, true negative values, and false-negative values could not be obtained directly or indirectly; (2) reviews, conference reports, case studies, and animal experiments; and (3) duplicate publications.

### Data extraction and literature quality assessment

Two researchers independently extracted the following data from the included literature: author, publication year, study population, study type, number of training sets and validation set (if there was no clear grouping in the text, we used the total sample for analysis). In addition, the number of deaths and survivors in the validation set were counted. For the studies for which a fourfold table could not be constructed, we calculated the number of deaths and survivors through the sample mortality rate. Regarding the machine learning models, each model included indicators, study locations, true-positive values, false-positive values, false-negative values, true-negative values, sensitivity values, and specificity values (for studies where there were multiple AI models in the validation set, we primarily analysed the model with the best overall performance). The QUADAS-2 tool was used to evaluate the quality of the included literature and the possibility of bias, and inconsistencies were resolved through by consulting a third investigator [11].

### Statistical analysis

Statistical analysis was performed using RevMan 5.3 for Mac, Stata 16.0 for Mac, and Meta-DiSc software. Threshold effect heterogeneity analysis was performed using Meta-DiSc 1.4 software, and the magnitude of heterogeneity was assessed by the I^2^ statistic. If the effect sizes of the studies were homogeneous, the fixed effects model was used; if there was heterogeneity, the random effects model was used. If there was obvious heterogeneity among the studies, the source of heterogeneity was further judged by sensitivity analysis, threshold effect, and nonthreshold effect analyses. The Sen merge, Spe merge, PLR merge, NLR merge, and DOR merge and their 95% confidence intervals (95% CI) were calculated separately by Stata 16.0 for Mac, the SROC curve was drawn, and the AUC was calculated. The Deeks test was used to evaluate the publication bias of the included studies. If P < 0.05, the included studies were considered to have publication bias.

## Results

### Literature search results and characteristics of the included studies

**Fig. 1:**
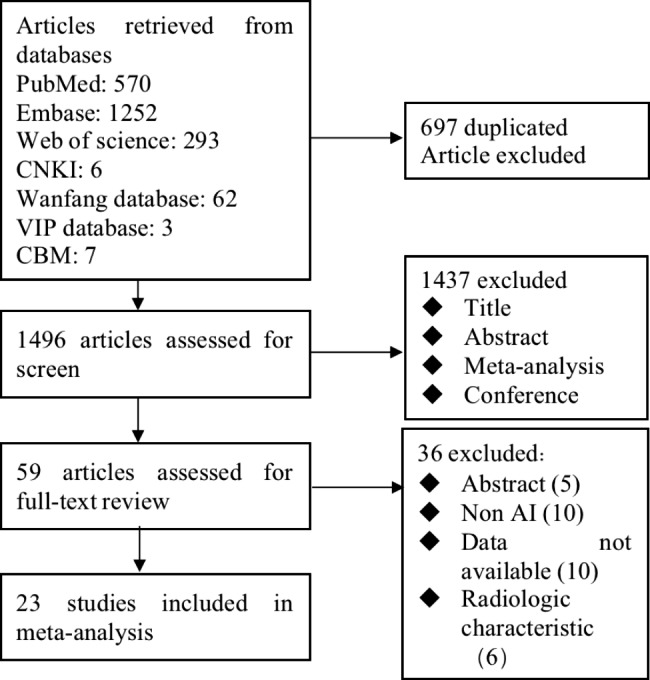
Literature screening flowchart

A total of 2193 studies were retrieved from the databases, and 0 studies were identified via manual search. After importing the studies into EndNote literature management software to check for duplicate studies, reading the abstracts and excluding relevant literature according to the exclusion criteria, 23 studies were finally included. The specific literature screening process and results are shown in Fig. 1. Table 1 shows the detailed characteristics of the 23 studies, which were conducted across a total of 12 countries and regions. Twenty-five AI models were used. There were 14 multicentre studies and 9 single-centre studies. Twenty-two studies were retrospective, and one study was cross-sectional. Fifteen studies distinguished between training and validation sets, and 5 studies did not explicitly mention grouping.

**Table 1:** characteristics of the included 23 studies

Author	Year	Selected time	Center	Study type	People	Mortality	Country	Model	Characteristic	Outcome	Total Patients, n	Training set/Validation set
Ashis Kumar Das [12]	2020	2020.1.20-2020.5.30	multicenterr	Retrospective	(KCDC2020)	2.10%	Korea	LR、SVM、KNN、RF、XGBoost	4	UA	3524	2819/705
Limin Yu [13]	2021	2020.2.1-2020.5.4	multicenter	Retrospective	ER departments of Beaumont Health	14.50%	Mexico	Cat boost	20	in-hospital mortality	1012	303、203
Fabiana Tezza [14]	2021	2020.3-2020.4	monocentric	Retrospective	Ospedali Riuniti Padova Sud	22.01%	Italy	RPART、SVM、GBM、RF	26	in-hospital mortality	341	UA
Alejandro Santos-Lozano [15]	2020	2020.2.28-2020.3.30	monocentric	Retrospective	large public Hospital of Madrid, Spain (Hospital 12 deOctubre)	20.38%	Spain	ANN	UA	in-hospital mortality	1369	UA
Prathamesh Parchure [16]	2020	2020.2.10-2020.4.7	multicenter	Retrospective	Mount Sinai Health System COVID-19 registry	17.00%	USA	RF	UA	in-hospital mortality	890	567/323
Rita Murri [17]	2021	2020.3.5-2021.2.5	monocentric	Retrospective	Fondazione Policlinico Gemelli	18.90%	Italy	LR	10	in-hospital mortality	2384	921/1463
Xiaoran Li [18]	2020	2020.2.7-2020.5.4	multicenter	Retrospective	Persons Under Investigation (PUI)	13.89%	USA	DNN	45	in-hospital mortality	1022	920/102
Sujoy Kar [19]	2021	2020.4-2020.6/2020.8-2020.10	multicenter	Retrospective	six Apollo Hospitals in India	8.54%	India	XGBoost	23	UA	2370	1393/977
Adam L. Booth [20]	2020	UA	multicenter	Retrospective	UA	10.80%	USA	SVM	5	UA	398	318/80
Emirena Garrafa [21]	2021	2020.3-2020.12	monocentric	Retrospective	ED and hospitalized at SCBH	18.15%	Italy	RF、GBM、LR	19	in-hospital mortality	2782	1776/676
Mohammad M. Banoei [22]	2021	2020.6-UA	multicenter	Retrospective	University of Miami Hospital, Miller School of Medicine, Miami, FL, USA,	12.40%	USA	SIMPLS	21	in-hospital mortality	250	172/78
Hoon Ko [23]	2020	2020.2-2020.7	multicenter	Retrospective	Wonkwang University Hospital (WKUH), Chonnam NationalUniversity Hospital (CNUH), and Samsung Medical Center (SMC) in Korea.	45.82%	Korea	XGBoost、ADABoost、RF、DNN、DNN + XGBoost、DNN + AdaBoost、DNN + RF	28	in-hospital mortality	467	361/106
Ju-Kuo Lin [24]	2021	UA	multicenter	Retrospective	Korea、Wuhan	35.97%	China	CNN、ANN、Forest、RF、Random tree、REPT Tree、BAYESNET、Naïve Bayes、LOGISTIC、SMO	30	in-hospital mortality	467	361/106
Ahmed Abdulaal [25]	2020	20202.2-2020.4.22	monocentric	Retrospective	A West London teaching hospital	23.00%	UK	ANN	22	in-hospital mortality	398	318/80
Fatemeh Moghaddam-Tabrizi [26]	2021	2020.2-2020.4	monocentric	Retrospective	Ghamar-Banihashem Hospital in Khoy district, Iran,	21.20%	Iran	RF	16	in-hospital mortality	401	UA
Abdulrhman Fahad Aljouie [27]	2021	2020.4.2-2020.6.18	multicenter	Retrospective	King Abdulaziz Medical City in Riyadh	8.99%	Saudi Arabia	RF	20	in-hospital mortality	1513	1212/301
Maleeha Naseem [28]	2021	2020.2-9	monocentric	cross-sectional study	Aga Khan University Hospital (AKUH), Karachi	10.71%	Pakistan	DNN、Deep-FLAIM、RF、KNN、SVC-RBF、DT、ABC、QDA	16	in-hospital mortality	1214	849/365
Khadijeh Moulaei [29]	2021	2020.3.5–9.22	multicenter	Retrospective	Shahid Mostafa Khomeini, Hazrat Valiasr hospital, and Imam Hossein hospital	23.53%	Iran	DT、Multilayer perceptron、KNN1、KNN2、KNN3、RF、SVM	16	UA	850	UA
Logan Ryan [30]	2020	2020.3.12-2020.4.12	monocentric	Retrospective	A community hospital	21.00%	USA	XGBoost	12	72 h mortality	114	UA
Kenji Ikemura [31]	2021	2020.3.1-2020.7.3	multicenter	Retrospective	Clinical Looking Glass (CLG)	25.20%	USA	XGBoost(48)、XGBoost(10)	48	30d mortality	4313	3468/845
Chi Peng [32]	2021	2020.2.5-2020.4.15	monocentric	Retrospective	Wuhan Huoshenshan Hospital or Guanggu Branch of Tongji Hospital of Tongji Medical College of Huazhong University of Science and Technology	2.08%	China	ANN、naive Bayes、LR、RF	49	in-hospital mortality	4804	3040/1764
Nicolás Munera [33]	2022	2020.3-2021.1	multicenterr	Retrospective	22 hospitals across eight Latin-American countries	23.70%	Colombia	RF	16	in-hospital mortality	2552	2378/69
Hongbing Peng [34]	2022	2020.1.11-2020.2.28	multicenterr	Retrospective	five hospitals in Hunan Province,China	3.22%	China	logistic regression	43	in-hospital mortality	62	UA

LR: logistic regression; SVM: support vector machine; RF: random forest; KNN: K Nearest Neighbors; GBM: Gradient boosting machine; Cat boost: categorical boosting; RPART: Recursive Partitioning and Regression Trees; SVM: Support Vector Machine; DNN: Deep Neural Network ; ANN: artificial neural network; XGBoost: eXtreme Gradient Boosting; ADABoost: Adaptive boosting; CNN: convolutional neural network; SVC-RBF: Support Vector Classifier - Radial Basis Function; DT: Decision Trees; ABC: Ada-Boost-Classifier; QDA: Quadratic Discriminant Analysis; UA:Unavailable

### Literature quality evaluation

According to the QUADAS-2 tool, the overall risk of bias in patient selection was unclear in 2 studies. All of the risks of bias in the index test and the reference standard test were low. All 23 studies had an unclear risk of bias for the flow and timing domains. In terms of overall concerns regarding applicability, only two studies had an unclear risk of bias in patient selection. The remaining concerns regarding applicability presented low risks (Supplementary Material 4).

### Results of the meta-analysis

#### Validation set (best model pooling)

**Fig. 2:**
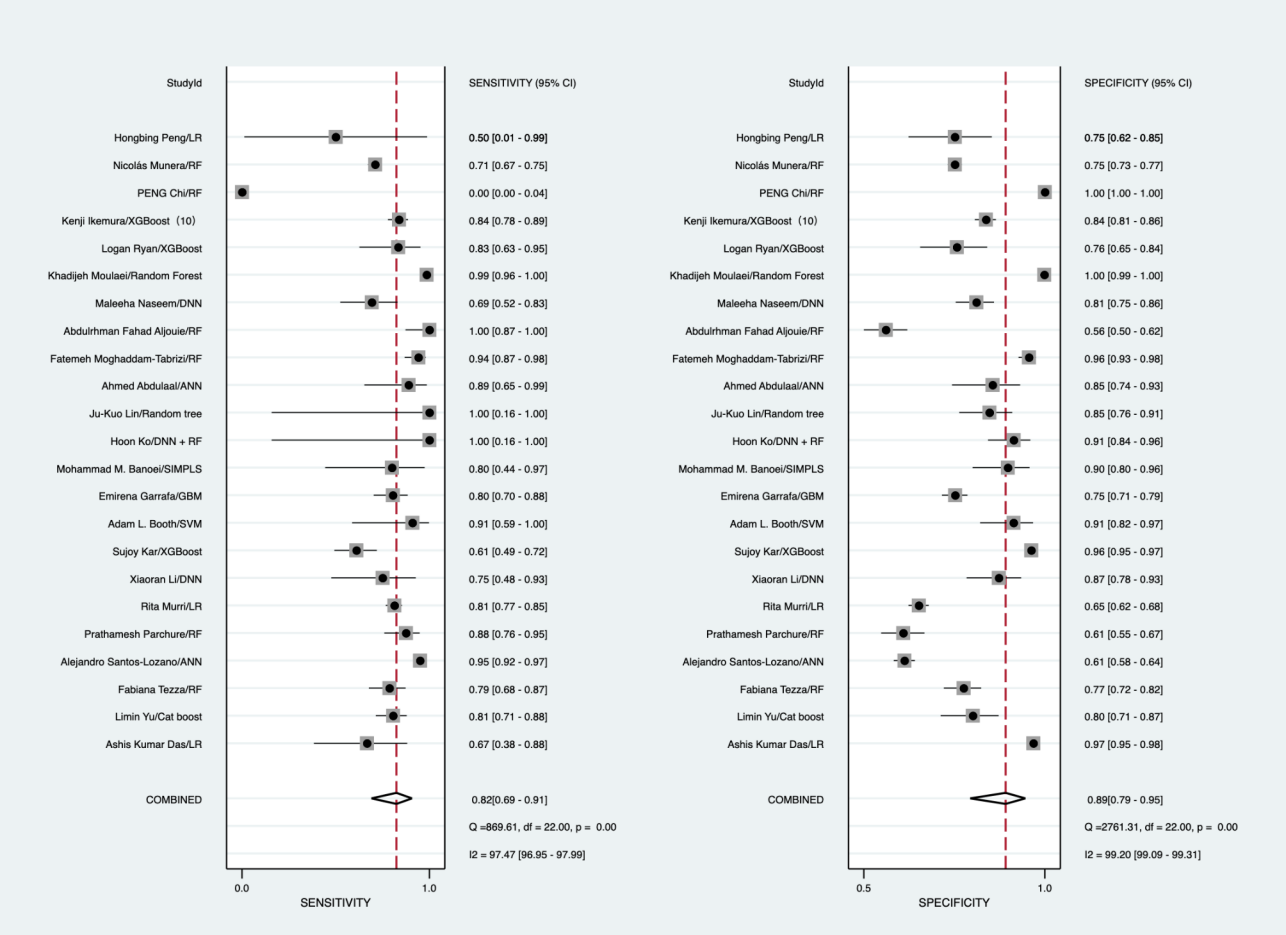
Forest plot of the pooled sensitivity and specificity

**Fig. 3:**
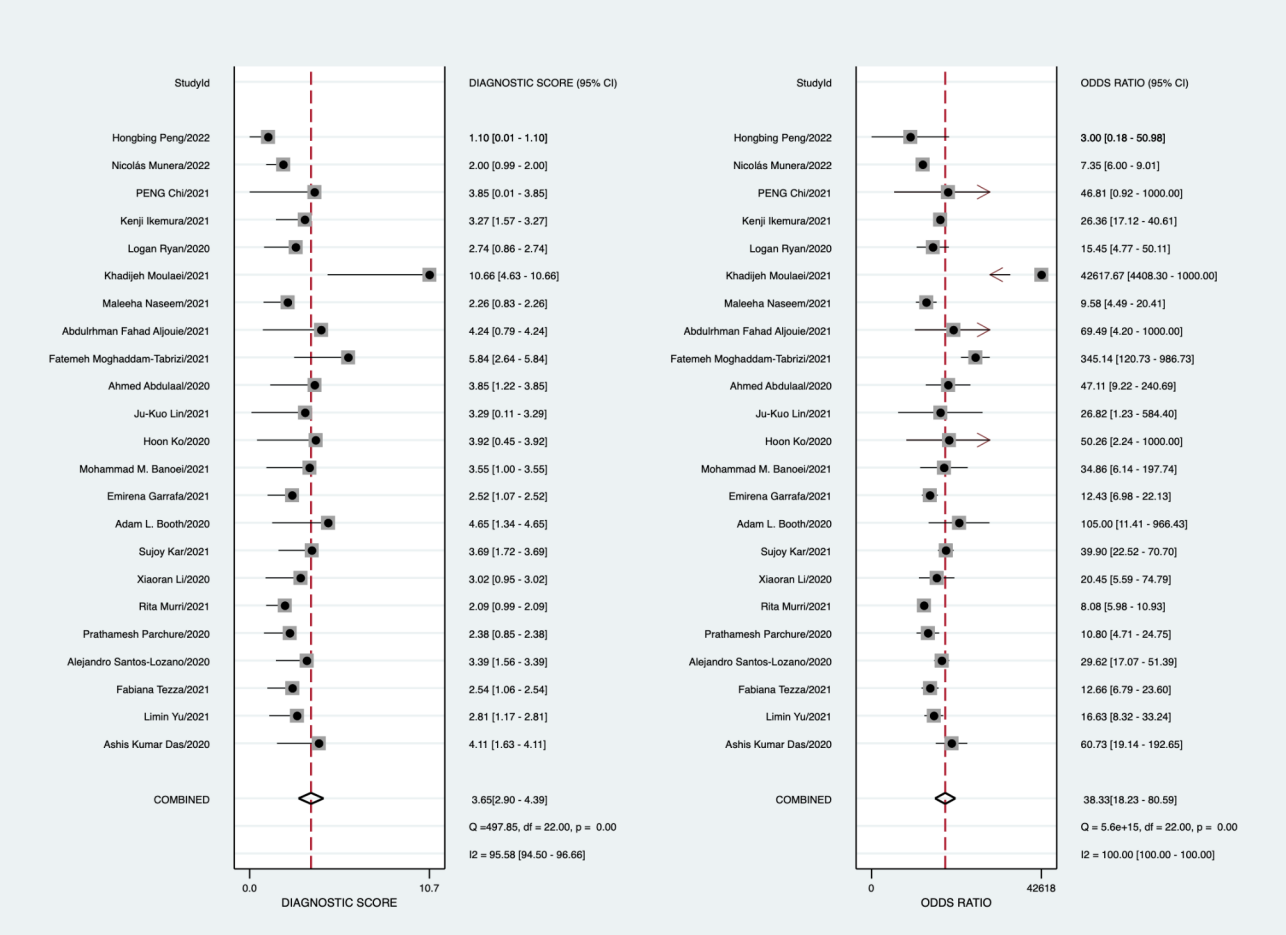
Forest plot of the pooled diagnostic odds ratio

**Fig. 4:**
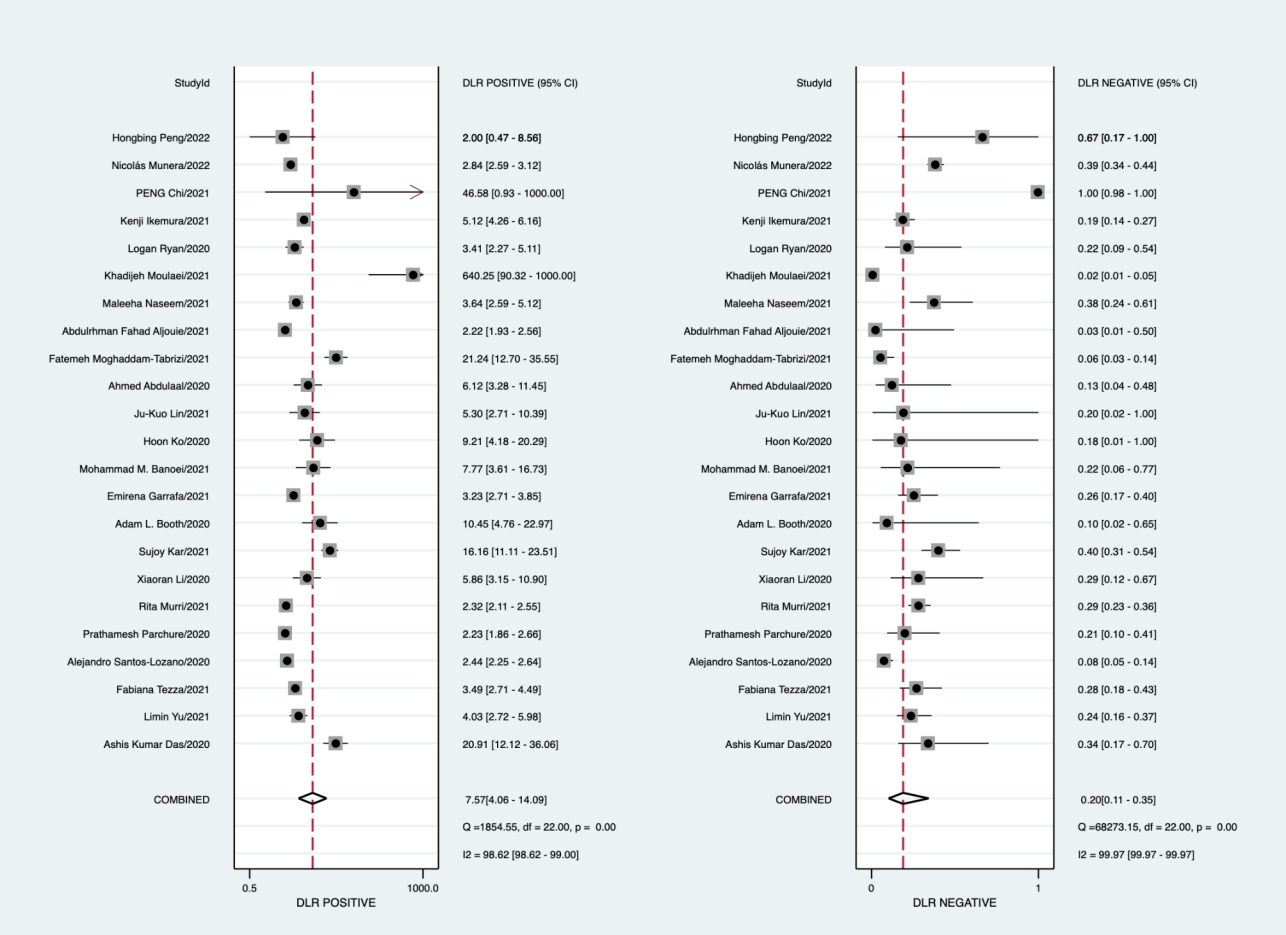
Forest plot of the pooled positive LR and negative LR

**Fig. 5:**
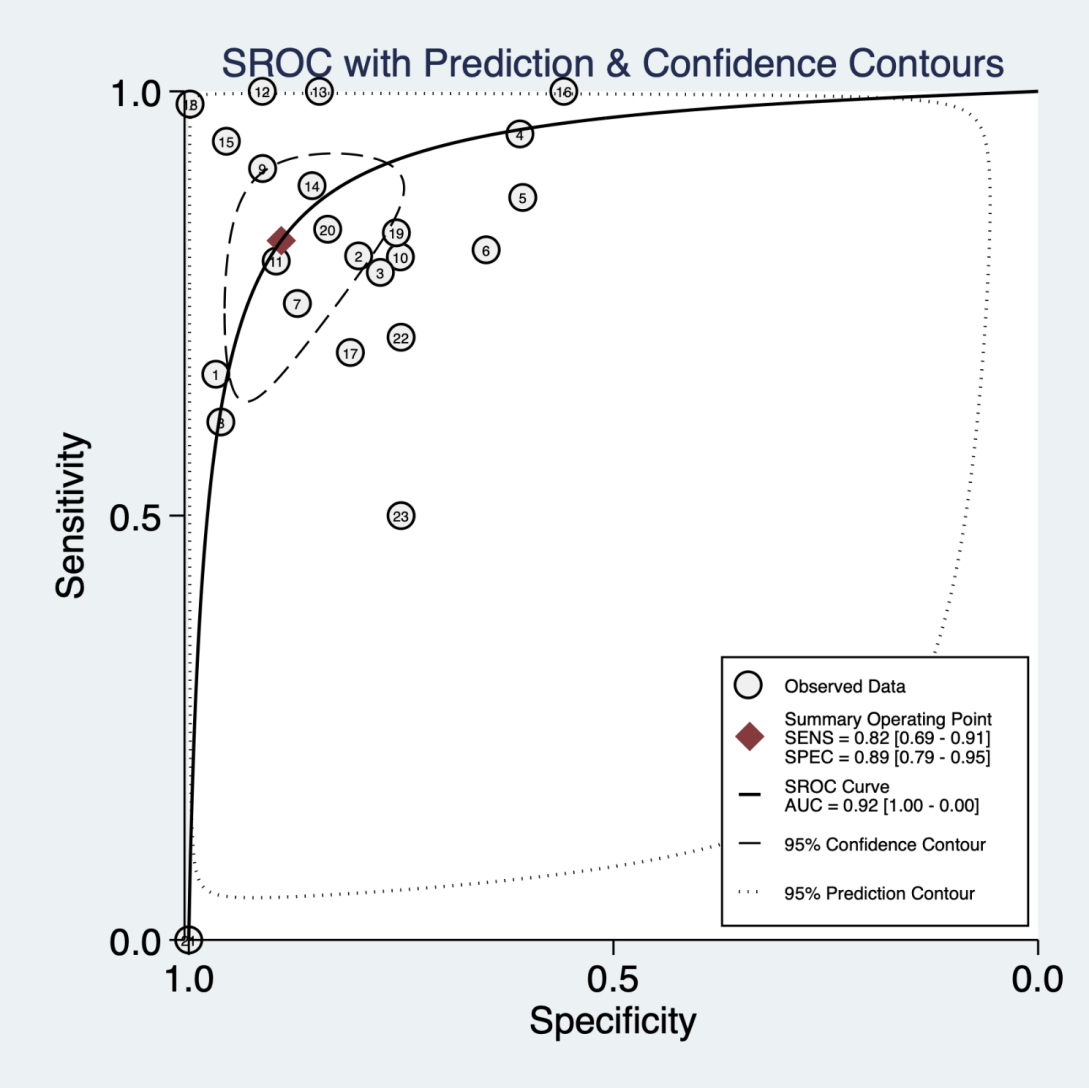
SROC of AI for the diagnosis of COVID-19 patient mortality

In the validation set, the best predictive model of the 23 studies assessed the performance of AI in predicting mortality in COVID-19 patients. The overall pooled AUROC was 0.92 [1.00, 0.00]. Additionally, the sensitivity, specificity, PLR, NLR, and diagnostic odds ratio were 0.82 [0.69, 0.91], 0.89 [0.79, 0.95], 7.57 [4.06, 14.09], 0.20 [0.11, 0.35], and 38.33 [18.23, 80.59], respectively (Figs. 2, 3, 4 and 5).

### Validation set (all model pooling)

In the validation set, a total of 25 models from 23 studies evaluated the performance of AI in predicting mortality in COVID-19 patients [12–34]. The overall pooled AUROC was 0.93 [1.00, 0.00]. Additionally, the sensitivity, specificity, PLR, NLR, and diagnostic odds ratio were 0.84 [0.78, 0.88], 0.89 [0.85, 0.92], 7.37 [5.38, 10.1], 0.18 [0.13, 0.25], and 40.15 [23.79, 67.74], respectively (Supplementary material 5; Figure S1-S4).

### Training set

In the training set, a total of 14 models from 5 studies evaluated the performance of AI in predicting mortality in COVID-19 patients. The overall pooled AUROC was 0.98 [0.96, 0.99]. Additionally, the sensitivity, specificity, PLR, NLR, and diagnostic odds ratio were 0.93 [0.87, 0.96], 0.94 [0.87, 0.97], 15.08 [6.89, 33.01], 0.07 [0.04, 0.14], and 202.41 [49.05, 835.20], respectively (Supplementary material 5; Figure S5-S8).

**Table 2:** Subgroup analyses of the performance of artificial intelligence in the diagnosis mortality in covid-19 patients

Subgroup	Study	Sensitivity	Specificity	PLR	NLR	DOR	AUC
**All combined**	23	0.82(0.69, 0.91)	0.89(0.79, 0.95)	7.60(4.1, 14.1)	0.20(0.11, 0.35)	38.00(18, 81)	0.92
**Model**							
RF	12	0.78(0.75–0.80)	0.81(0.80–0.82)	5.00(3.41–7.33)	0.21(0.12–0.37)	31.59(13.06–76.40)	0.92
XGBoost	5	0.83(0.79–0.86)	0.87(0.86–0.89)	6.02(3.75–9.65)	0.24(0.14–0.41)	29.82(22.72–39.16)	0.91
LR	6	0.80(0.77–0.84)	0.85(0.84–0.85)	4.32(1.74–10.74)	0.31(0.25–0.38)	12.94(3.52–47.51)	0.86
SVM	4	0.94(0.91–0.97)	0.90(0.88–0.91)	9.48(1.64–57.74)	0.11(0.02–0.47)	90.33(9.84–828.9)	0.98
ANN	4	0.91(0.88–0.94)	0.88(0.88–0.89)	4.85(1.31–17.96)	0.14(0.07–0.26)	43.49(16.88-112.07)	0.94
DNN	3	0.70(0.57–0.82)	0.85(0.81–0.88)	4.12(3.07–5.52)	0.37(0.25–0.55)	11.56(6.11–21.86)	0.83
KNN	3	0.91(0.89–0.93)	0.96(0.95–0.97)	24.63(5.49-110.58)	0.12(0.02–0.85)	231.65(15.92-3369.65)	0.98
GBM	2	0.83(0.76–0.88)	0.72(0.69–0.75)	2.86(2.25–3.65)	0.24(0.17–0.34)	11.98(7.7-18.65)	0.50
DT	2	0.89(0.84–0.92)	0.95(0.94–0.97)	13.17(1.38-125.62)	0.12(0-61.10)	112.39(0.4-31797.54)	0.50
**Mortality**							
0–10%	5	0.67(0.31–0.90)	0.97(0.90–0.99)	19.60(11-34.9)	0.34(0.13–0.91)	58.00(35–95)	0.96
10–20%	8	0.72(0.69–0.75)	0.73(0.72–0.74)	3.62(2.76–4.75)	0.41(0.32–0.53)	8.61(6.46–11.47)	0.80
> 20%	10	0.90(0.85–0.94)	0.87(0.80–0.92)	7.10(4.4–11.3)	0.11(0.07–0.19)	63.00(26–151)	0.95
**Center**							
multicenter	14	0.87(0.86–0.88)	0.89(0.88–0.89)	7.54(5.35–10.62)	0.18(0.12–0.26)	52.58(27.35–101.10)	0.93
monocentric	9	0.77(0.75–0.79)	0.87(0.87–0.88)	4.60(3.18–6.64)	0.33(0.25–0.43)	14.85(9.30–23.70)	0.88
**People**							
Asia	10	0.87(0.86–0.88)	0.95(0.95–0.95)	9.59(5.92–15.55)	0.20(0.13–0.31)	56.94(28.76-112.74)	0.94
Non Asia	13	0.52(0.51–0.54)	0.64(0.63–0.65)	3.09(2.08–4.59)	0.26(0.14–0.48)	12.30(3.83–39.45)	0.84
**Outcome**							
In-hospital mortality	17	0.76(0.75–0.78)	0.85(0.85–0.86)	4.14(3.28–5.24)	0.33(0.26–0.40)	14.50(10.28–20.45)	0.85

Note: LR: logistic regression; SVM: support vector machine; RF: random forest; KNN: K Nearest Neighbors; GBM: Gradient boosting machine; SVM: Support Vector Machine; DNN: Deep Neural Network ; ANN: artificial neural network; XGBoost: eXtreme Gradient Boosting;

### Subgroup Analysis results

1. In the subgroup analysis of each AI model, we found that the areas under the summary receiver operating characteristic (SROC) curves of KNN, SVM, ANN, RF, XGBoost, LR, DNN, GBM, and DT were 0.98, 0.98, 0.94 0.92, 0.91, 0.86, 0.83, 0.50, and 0.50, respectively. Subgroup analysis was not possible due to the small number of studies that included other models. (Table 2)
2. In the subgroup analysis of mortality, the areas under the summary receiver operating characteristic (SROC) curve of 0-10%, 10-20%, and > 20% were 0.96, 0.80, and 0.95, respectively. (Table 2)
3. In the subgroup analysis of the study centres, the areas under the summary receiver operating characteristic (SROC) curves of the multicentre and single-centre studies were 0.93 and 0.88, respectively. (Table 2)
4. In the regional subgroup analysis, the area under the summary receiver operating characteristic (SROC) curve for Asian and non-Asian regions was 0.94 and 0.84, respectively. (Table 2)
5. In subgroup analyses with in-hospital mortality as the outcome measure, the overall pooled AUROC was 0.85. Additionally, the sensitivity, specificity, PLR, NLR, and diagnostic odds ratio were 0.76 [0.75, 0.78], 0.85 [0.85, 0.86], 4.14 [3.28, 5.24], 0.33 [0.26, 0.40], and 14.50 [10.28, 20.45], respectively. (Table 2)

### Heterogeneity analysis

The results of the heterogeneity test found significant heterogeneity among the studies; a random effects model was used for meta-analysis. Spearman’s correlation coefficient for log sensitivity and log specificity was 0.054 (p = 0.81), suggesting no threshold effect. After excluding the threshold effect heterogeneity, we conducted a sensitivity analysis. After removing each study in turn, the results showed no significant difference between the combined effect size and the total combined effect after removing a single study, indicating that the results were stable and reliable (Supplementary material 5. Figure S9).

### Publication bias detection

**Fig. 6:**
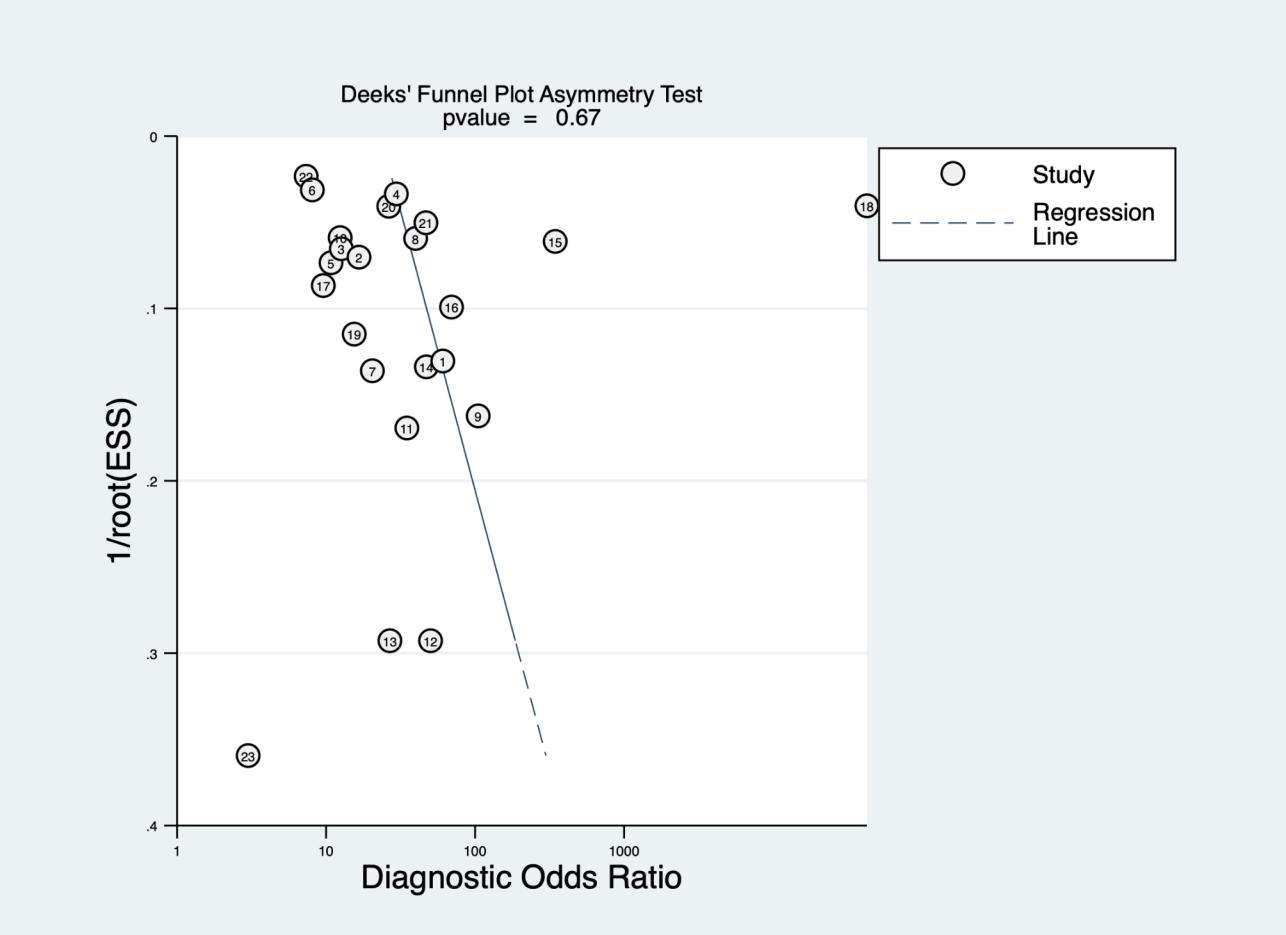
Funnel plot of studies included in the meta-analysis

The results of the Deeks test showed that p = 0.67 (p > 0.05), indicating no publication bias in the included literature (Fig. 6).

## Discussion

COVID-19 continues to spread, and global epidemic prevention work has basically brought about the stage of “normalization”. However, as new strains continue to emerge, the death toll from COVID-19 continues to climb, putting enormous pressure on the global health care system. At present, the MEWS [35], APACHE [36], Simplified Acute Physiology Score (SAPS II) [37], Sepsis-related Organ Failure Assessment (SOFA) [38], and Rapid SOFA Score [39] are often used clinically as tools to estimate the death rate of COVID-19, but these scales fail to provide accurate and reliable predictions of mortality in patients with COVID-19 and have limited specificity and sensitivity. Therefore, finding predictive tools with higher diagnostic accuracy is crucial for improving the prognosis of patients with COVID-19.

The advent of AI has generated rapid progress in the diagnosis and prognosis of COVID-19. AI has gradually attracted the attention of clinicians with its large, advanced algorithms in image recognition, data analysis, decision assistance, and other aspects [40, 41]. Therefore, in this study, we conducted the first meta-analysis of algorithm performance in AI prediction of COVID-19 patient mortality.

In the literature included in this meta-analysis, the dataset used by the AI model was divided into a training set and a validation set. The sensitivity of the training set was 0.93 [0.87, 0.96], the specificity was 0.94 [0.87, 0.97], and the AUC was 0.98 [0.96, 0.99]. The sensitivity of the validation set was 0.84 [0.78, 0.88], the specificity was 0.89 [0.85, 0.92], and the AUC was 0.93 [1.00, 0.00]. Compared with the training set, the diagnostic performance of the validation set was slightly reduced, but the difference was not obvious, and the AUC value was still high. This indicates that AI predicts the mortality of patients with high accuracy, and the results are repeatable and reliable.

A subgroup analysis was performed according to different AI models from the studies we included. The results show that among the various models, KNN, SVM, and ANN performed the best, and their AUCs were 0.98, 0.98, and 0.94, respectively. This seems to contradict the general pattern in machine learning: XGBoost tends to perform better than the KNN and SVM models under the same conditions. By analysing the experimental process of comparative literature, we explain this phenomenon from two perspectives. First, from the perspective of data characteristics, the input data in the above research are all low-dimensional (the maximum number of features is 48). SVM and KNN are suitable for dealing with such problems. In contrast, XGBOOST is better suited to handle more complex relationships between data features and targets, and overfitting problems can occur in the case of fewer features. Second, the difference in data quality affects the model performance. The original data used by SVM and KNN do not have the problem of missing data, while the original data of other models do have the problem of missing data, and the processing methods are too simple, such as simple mean and median replacement. As a result, the latter prediction is less than satisfactory. For example, in Prathamesh Parchure’s study, the proportion of missing data ranges from 53.5 to 89.0%, and more than 60% of missing data will make it difficult for any data interpolation method to obtain satisfactory results [42]. An C et al. and Sun L et al. also proposed that the SVM algorithm has high sensitivity and specificity for predicting the mortality of COVID-19 patients with high accuracy and stability [43, 44].

In addition, these AI models predict mortality based on all or part of the clinical characteristics, including demographics (e.g., age, sex, ethnicity), comorbidities (e.g., diabetes, heart disease), symptoms (e.g., cough, fever), vital signs (e.g., heart rate, oxygen saturation), laboratory tests (e.g., blood glucose, creatinine, haemoglobin), imaging measures (e.g., X-ray), and disease treatment and clinical course (e.g., artificial ventilation, length of hospital stay, medications). Algorithms with high AUC values use more predictors. We found that the most commonly adopted predictors of mortality were age, C-reactive protein, and comorbidities. Previously published clinical studies have shown that age, C-reactive protein, and comorbidities play important roles in predicting mortality in patients with COVID-19 [43, 45–47].

A previous study by Escobar GJ et al. showed that race was not associated with mortality after COVID-19 infection [48]. We divided the included studies into two groups, Asian and non-Asian, for subgroup analysis and found that the AUC value of the Asian group was 0.94, and the AUC value of the non-Asian group was 0.84. The AI model performed better in the diagnosis of the Asian group. This suggests that when our AI model is extended to people in different regions, the stability of prediction may change to a certain extent due to differences in local medical care levels and new disease prevention and control measures. This suggests that when the model is popularized and applied, it should be adjusted and calibrated according to changes in factors such as regions to improve the accuracy of the diagnosis of the target population.

To further explore the heterogeneity of the studies, we used sensitivity analysis to remove each study one by one, and the results did not change significantly. There was no significant difference between the combined effect size and the total combined effect size after removing a single study with large heterogeneity. The results are stable and reliable.

AI prediction of the mortality of COVID-19 patients can help clinicians make decisions on the length of hospital stay and whether to upgrade according to the risk stratification of predicted patient mortality. In the context of the COVID-19 pandemic, especially in the case of ventilator shortages, it can help medical resource management teams allocate resources and optimize patient management [13, 28]. At present, pathology and radiotherapy guidelines for patients with COVID-19 need to be supplemented. AI model prediction of mortality in COVID-19 patients can help pathologists and radiologists more accurately interpret pathological imaging results to aid diagnosis and treatment. COVID-19 patients who receive AI to accurately predict mortality can also decide whether to discharge or receive palliative care according to the level of their own prediction results to make more appropriate decisions [16].

Our research also has certain limitations. First, the number of studies we included is relatively limited. Due to the lack of relevant articles on AI models based on imaging features, we did not include them in the analysis. We hope that more studies will be conducted in the future with the ability to develop and validate models with imaging features. Second, there were as many as 25 AI models in our included articles, which we believe may be a major source of heterogeneity. In our included studies, the baseline variables (e.g., demographic characteristics, vital signs, comorbidities, laboratory tests) included in each model differed to some extent, which may also be a source of some of the heterogeneity (Supplementary material 6). In addition, the use of “English” as the sole language for searches leads to potential bias due to the large number of studies in other languages involved in COVID-19 studies. Finally, and most importantly, none of our included studies addressed the vaccination status of the included population, which has a strong impact on mortality in COVID-19 patients. It is hoped that future studies will include vaccines as a parameter in AI models to improve their application value in COVID-19 patients.

## Conclusion

Compared with traditional COVID-19 mortality prediction tools, the AI model has higher accuracy in predicting the mortality of COVID-19 patients, better predictive performance, and higher prognostic value. Among them, KNN, SVM, RF, ANN, XGBoost, and other models have higher accuracy.

### Electronic supplementary material

Below is the link to the electronic supplementary material.

Supplementary Material 1：PRISMA-DTA

Supplementary Material 2：PRISMA-DTA for Abstracts

Supplementary Material 3：Detailed retrieval strategy

Supplementary Material 4：AI Model Detailed Predictors

Supplementary Material 5：Table S1-S2, Figure S1-S9

Supplementary Material 6：Risk of bias assessments

## Data Availability

All data supporting the conclusions presented in this article are included in this published article.

## Abbreviations

LR: logistic regression
SVM: support vector machine
RF: random forest
KNN: K Nearest Neighbors
GBM: Gradient boosting machine
Cat boost: categorical boosting
RPART: Recursive Partitioning and Regression Trees
SVM: Support Vector Machine
DNN: Deep Neural Network
ANN: artificial neural network
XGBoost: eXtreme Gradient Boosting
ADABoost: Adaptive boosting
CNN: convolutional neural network
SVC-RBF: Support Vector Classifier - Radial Basis Function
DT: Decision Trees
ABC: Ada-Boost-Classifier
QDA: Quadratic Discriminant Analysis
AI: Artificial intelligence
QUADAS-2: Quality Assessment of Diagnostic Accuracy Studies-2
CI: confidence interval
SEN: sensitivity
SPE: specificity
NLR: negative likelihood ratio
PLR: positive likelihood ratio
DOR: diagnostic odds ratio
SEN: sensitivity
SPE: specificity
TP: true positive
FP: false positive
TN: true negative
FN: false negative
AUC: area under the curve
SROC: summary receiver operating characteristic
