# Supplementary material for: The accuracy of artificial intelligence in predicting COVID-19 patient mortality: a systematic review and meta-analysis
Source: BMC Med Inform Decis Mak. 2023 Aug 9;23:155. doi: 10.1186/s12911-023-02256-7 (PMC10410953; doi:10.1186/s12911-023-02256-7)
Supplement: Supplementary file 1 — Supplementary Material 1：PRISMA-DTA [file 12911_2023_2256_MOESM1_ESM.doc]

| **Section/topic** | **#** | **PRISMA-DTA Checklist Item** | **Reported on page #** |
| --- | --- | --- | --- |
| **TITLE / ABSTRACT** | | |  |
| Title | 1 | The accuracy of artificial intelligence in predicting COVID-19 patient mortality: a systematic review and meta-analysis | 1 |
| Abstract | 2 | Abstract: See PRISMA-DTA for abstracts. | / |
| INTRODUCTION | | |  |
| Rationale | 3 | Effective and accurate outcome prediction and effective and classified management of patients are increasingly important. However, there is still a lack of tools to predict the risk of death in COVID-19 patients. AI algorithms represented by deep learning are gradually being applied in various medical fields and have achieved good diagnostic performance | 4 |
| Clinical role of index test | D1 | / |  |
| Objectives | 4 | The purpose of this paper is to systematically evaluate the application value of artificial intelligence in predicting the mortality of COVID-19 patients. | 2 |
| METHODS | | |  |
| Protocol and registration | 5 | Prospero：CRD42022315158 |  |
| Eligibility criteria | 6 | Inclusion criteria: 1) The study must be in English and be peer-reviewed; 2) Provide results from machine learning algorithms and predictions of mortality in COVID-19 patients; 3) Data must be complete with sample size, sensitivity, and specificity; 4) provide the total number of patients with COVID-19; 5) the study subjects were patients who were confirmed positive for COVID-19 by reverse transcription-polymerase chain reaction (RT–PCR); 6) clearly described the Machine learning models and predictors used in the predictions, 7) A clear overview of the sources of the datasets used in the study. | 5-6 |
| Information sources | 7 | Computer searches of the PubMed, Embase and Web of Science databases. The retrieval time is from the establishment of the database to January 2022. | 5 |
| Search | 8 | Search by a combination of subject headings and free words. Search terms included "Artificial Intelligence", "Machine Intelligence", "Machine learning", "AI", "deep learning", "random forest", "Mortality", "diagnosis", "sars-cov-2", and "covid" -19" | 5 |
| Study selection | 9 | Two reviewers independently screened the literature, extracted data and evaluated the included studies according to the inclusion criteria,exclusion criteria and methodological quality. In case of disagreement, discuss and resolve or hand over to a third party assist in ruling. | 6 |
| Data collection process | 10 | Two researchers extract the data according to the designed data extraction table, and finally cross-check the extraction situation. If there is any difference, it will be resolved through discussion and negotiation. | 6 |
| Definitions for data extraction | 11 | Two researchers independently extracted the following information about the included literature: author, publication year, study population, study type, number of training sets and validation set (if there was no clear grouping in the text, we used the total sample for analysis). The number of deaths or survivors in the validation set and the literature for which the four-table table cannot be obtained, we will calculate the four-table table through the sample mortality rate), machine learning model, each model focuses on the inclusion of indicators, study locations, true positive values, and false positives value, false negative value, true negative value, and sensitivity and specificity (for studies where there are multiple AI models in the validation set, we mainly analyze the model with the best overall performance). The QUADAS-2 was used to evaluate the quality of the included literature and the possibility of bias if inconsistencies were resolved through consultation or discussion with a third investigator. | 6 |
| Risk of bias and applicability | 12 | Assess the quality of the Diagnostic Accuracy Studies-2 (QUADAS-2) checklist. | 6 |
| Diagnostic accuracy measures | 13 | ROC curve analysis was used for the included studies to calculate the combined sensitivity, specificity, positive likelihood ratio, negative likelihood ratio, diagnostic odds ratio, and area under the curve (AUC). All results were expressed with 95% CI. | 7 |
| Synthesis of results | 14 | Statistical analysis was performed using RevMan 5.3 for mac, Stata 16.0 for mac and metadisc software. Threshold effect heterogeneity analysis was performed using Meta Disc 1.4 software, and the magnitude of heterogeneity was assessed by I2. If the effect sizes of the studies were homogeneous, the fixed-effects model was used; if there was heterogeneity, the random-effects model was used. If there was obvious heterogeneity among the studies, the source of heterogeneity was further judged by sensitivity analysis, threshold effect and nonthreshold effect analysis. The Sen merge, Spe merge, PLR merge, NLR merge, DOR merge and their 95% confidence intervals (95% CI) were calculated separately by Stata 16.0 for mac, the SROC curve was drawn, and the AUC was calculated. At the same time, the Deeks test was used to evaluate the publication bias of the included literature. If P<0.05, the included literature was considered to have publication bias. | 7 |

Page 1 of 2

| Section/topic | # | PRISMA-DTA Checklist Item | Reported on page # |
| --- | --- | --- | --- |
| Meta-analysis | D2 | Reference no. 14 |  |
| Additional analyses | 16 | We performed subgroup analyses based on different AI models（KNN, SVM, RF, ANN, XGBoost, LR, DNN, GBM, and DT）, different regions, mortality rates, and sample sources | 9 |
| RESULTS | | |  |
| Study selection | 17 | A total of 1443 studies were initially searched, and 0 studies were manually searched. After importing the endnote literature management software to check the duplication and reading the abstracts and excluding relevant literature according to the exclusion criteria, 20 studies were finally included. | 7 |
| Study characteristics | 18 | The specific literature screening process and results are shown in Figure 1. Table 1 shows the detailed characteristics of 20 studies, involving a total of 11 countries and regions, 25 cumulative AI models, 12 multicenter studies, and 8 single-center studies. Nineteen studies were retrospective, one was cross-sectional, 15 studies clearly distinguished training and validation sets, and 5 studies did not explicitly mention grouping. | 7-8 |
| Risk of bias and applicability | 19 | According to the QUADAS-2 tool, the overall risk of bias in patient selection was unclear in 2 (10%) studies. All of the risks of bias in the index test and the reference standard test were low. Flow and timing had all 20 studies with unclear risk of bias. In Overall applicability concerns. Only one article had an unclear risk of bias in patient selection. The remaining applicability concerns are low risk (Figure 2, 3). | 8 |
| Results of individual studies | 20 | The research results are displayed in the form of tables and forest diagrams |  |
| Synthesis of results | 21 | Validation set (best model pooling)  In the validation set, the best predictive model of 20 studies assessed AI's performance in predicting mortality in COVID-19 patients. The overall pooled AUROC for identifying sepsis patients was 0.93 [0.90, 0.95]. Additionally, the sensitivity, specificity, PLR, NLR, and diagnostic odds ratio were 0.87 [0.81, 0.91], 0.87 [0.79, 0.92], 6.5 [4.0, 10.6], 0.15 [0.10, 0.23], and 42 [20, 90], respectively (Figure 4-7).  Validation set (all models pooling)  In the validation set, a total of 25 models from 20 studies evaluated the performance of AI in predicting mortality in COVID-19 patients. The overall pooled AUROC for identifying sepsis patients was 0.93 [0.90, 0.95]. Additionally, the sensitivity, specificity, PLR, NLR, and diagnostic odds ratio were 0.84 [0.78, 0.89], 0.88 [0.84, 0.91], 6.9 [5.0, 9.5], 0.18 [0.13, 0.26], and 38 [22, 65], respectively (Figure S1-S4).  Training set  In the training set, a total of 14 models from 5 studies evaluated the performance of AI in predicting mortality in COVID-19 patients. The overall pooled AUROC for identifying sepsis patients was 0.98 [0.96, 0.99]. Additionally, the sensitivity, specificity, PLR, NLR, and diagnostic odds ratio were 0.93 [0.87, 0.96], 0.94 [0.87, 0.97], 15.08 [6.89, 33.01], 0.07 [0.04, 0.14], and 202.41 [49.05, 835.20], respectively (Figure S5-S8). | 8-9 |
| Additional analysis | 23 | **Subgroup Analysis Results**  1). In the subgroup analysis of each artificial intelligence model, we found that the area under the comprehensive receiver operating characteristic (SROC) curve of KNN, SVM, RF, ANN, XGBoost, LR, DNN, GBM, and DT was 0.9846., 0.9803, 0.932, 0.926, 0.9108, 0.8533, 0.8316, 0.50, 0.50. Subgroup analysis was not possible due to the small number of studies of other models.  2). In the subgroup analysis of mortality, the areas under the comprehensive receiver operating characteristic (SROC) curve of 0%-10%, 10%-20%, and >20% were 0.9444, 0.8077, and 0.9424, respectively. (Table 2)  3). In the subgroup analysis of the study centers, the areas under the composite receiver operating characteristic (SROC) curves of the multicenter and single-center studies were 0.9393 and 0.8397, respectively. (Table 2)  4). In the regional subgroup analysis, the area under the composite receiver operating characteristic (SROC) curve for Asian and non-Asian regions was 0.9343 and 0.8660, respectively. (Table 2)  **Heterogeneity Analysis**  The results of the heterogeneity test found that there was significant heterogeneity among the studies, so a random-effects model was used for meta-analysis. The Spearman correlation coefficient for log sensitivity and 1-log specificity was 0.163 p = 0.493, suggesting no threshold effect. After excluding the threshold effect heterogeneity, we conducted a sensitivity analysis. After removing each study in turn, the results showed that there was no significant difference between the combined effect size and the total combined effect after removing a single study, indicating that the results were stable and reliable. (Figure S9)  **Publication bias detection**  The results of the Deeks test showed that p=0.81 (p>0.05), indicating that there was no publication bias in the included literature. (Figure 8) | 9-10 |
| DISCUSSION | | |  |
| Summary of evidence | 24 | Of the 1443 studies, 20 studies met the inclusion criteria, accumulating 25 AI models, of which 15 studies explicitly mentioned training and test sets, and 5 studies did not explicitly mention grouping. In the training set, the pooled sensitivity was 0.93 [0.87, 0.96], the pooled specificity was 0.94 [0.87, 0.97], and the area under the ROC curve was 0.98 [0.96, 0.99]. In the validation set, the pooled sensitivity was 0.87 [0.81, 0.91], the pooled specificity was 0.87 [0.79, 0.92], and the area under the ROC curve was 0.93 [0.90, 0.95]. In the subgroup analysis, the areas under the comprehensive receiver operating characteristic (SROC) curve of the artificial intelligence models KNN, SVM, RF, ANN, and XGBoost were 0.9846, 0.9803, 0.932, 0.926, and 0.9108, respectively. The Deeks funnel plot showed that there was no significant difference in the publication bias of this study (P>0.05). | 7-10 |
| Limitations | 25 | First, the number of studies we included is relatively limited. Due to the lack of relevant articles on artificial intelligence models based on imaging features, we did not include them in the analysis. We hope that more studies will be conducted in the future. Ability to develop and validate models with imaging features. Second, there were as many as 25 AI models in our included articles, which we believe may be a major source of heterogeneity. Finally, in our included literature, baseline variables (e.g., demographic characteristics, vital signs, comorbidities, laboratory tests) included in each model differed to some extent, which may also be a source of some of the heterogeneity | 9 |
| Conclusions | 26 | Compared with traditional COVID-19 mortality screening tools, the artificial intelligence model has high accuracy in predicting the mortality of COVID-19 patients, better prediction performance, and higher prognostic value. Among them, KNN, SVM, RF, ANN, XGBoost and other models have higher accuracy. | 10 |
| FUNDING | | |  |
| Funding | 27 | This work was supported by the Technology Department and Natural Science Foundation for Distinguished Young Scholars of Heilongjiang Province (JQ2021H003). | 15 |

*Adapted From:*  McInnes MDF, Moher D, Thombs BD, McGrath TA, Bossuyt PM, The PRISMA-DTA Group (2018). Preferred Reporting Items for a Systematic Review and Meta-analysis of Diagnostic Test Accuracy Studies: The PRISMA-DTA Statement. JAMA. 2018 Jan 23;319(4):388-396. doi: 10.1001/jama.2017.19163.

For more information, visit: **www.prisma-statement.org**.

Page 2 of 2
