# Supplementary material for: The accuracy of artificial intelligence in predicting COVID-19 patient mortality: a systematic review and meta-analysis
Source: BMC Med Inform Decis Mak. 2023 Aug 9;23:155. doi: 10.1186/s12911-023-02256-7 (PMC10410953; doi:10.1186/s12911-023-02256-7)
Supplement: Supplementary file 2 — Supplementary Material 2：PRISMA-DTA for Abstracts [file 12911_2023_2256_MOESM2_ESM.doc]

| **Section/topic** | **#** | **PRISMA-DTA for Abstracts Checklist item** | **Reported on page #** |
| --- | --- | --- | --- |
| **TITLE and PURPOSE** | | |  |
| Title | 1 | The accuracy of artificial intelligence in predicting COVID-19 patient mortality: a systematic review and meta-analysis | 1 |
| Objectives | 2 | The purpose of this paper is to systematically evaluate the application value of artificial intelligence in predicting the mortality of COVID-19 patients. | 2 |
| METHODS | | |  |
| Eligibility criteria | 3 | Inclusion criteria: 1) The study must be in English and be peer-reviewed; 2) Provide results from machine learning algorithms and predictions of mortality in COVID-19 patients; 3) Data must be complete with sample size, sensitivity, and specificity; 4) provide the total number of patients with COVID-19; 5) the study subjects were patients who were confirmed positive for COVID-19 by reverse transcription-polymerase chain reaction (RT–PCR); 6) clearly described the Machine learning models and predictors used in the predictions, 7) A clear overview of the sources of the datasets used in the study. | 5-6 |
| Information sources | 4 | Computer searches of the PubMed, Embase and Web of Science databases. The retrieval time is from the establishment of the database to January 2022. | 5 |
| Risk of bias & applicability | 5 | Assess the quality of the Diagnostic Accuracy Studies-2 (QUADAS-2) checklist. | 6 |
| Synthesis of results | A1 | Statistical analysis was performed using RevMan 5.3 for mac, Stata 16.0 for mac and metadisc software. Threshold effect heterogeneity analysis was performed using Meta Disc 1.4 software, and the magnitude of heterogeneity was assessed by I2. If the effect sizes of the studies were homogeneous, the fixed-effects model was used; if there was heterogeneity, the random-effects model was used. If there was obvious heterogeneity among the studies, the source of heterogeneity was further judged by sensitivity analysis, threshold effect and nonthreshold effect analysis. The Sen merge, Spe merge, PLR merge, NLR merge, DOR merge and their 95% confidence intervals (95% CI) were calculated separately by Stata 16.0 for mac, the SROC curve was drawn, and the AUC was calculated. At the same time, the Deeks test was used to evaluate the publication bias of the included literature. If P<0.05, the included literature was considered to have publication bias. | 7 |
| RESULTS | | |  |
| Included studies | 6 | A total of 1443 studies were initially searched, and 0 studies were manually searched. After importing the endnote literature management software to check the duplication and reading the abstracts and excluding relevant literature according to the exclusion criteria, 20 studies were finally included. | 7 |
| Synthesis of results | 7 | Validation set (best model pooling)  In the validation set, the best predictive model of 20 studies assessed AI's performance in predicting mortality in COVID-19 patients. The overall pooled AUROC for identifying sepsis patients was 0.93 [0.90, 0.95]. Additionally, the sensitivity, specificity, PLR, NLR, and diagnostic odds ratio were 0.87 [0.81, 0.91], 0.87 [0.79, 0.92], 6.5 [4.0, 10.6], 0.15 [0.10, 0.23], and 42 [20, 90], respectively (Figure 4-7).  Validation set (all models pooling)  In the validation set, a total of 25 models from 20 studies evaluated the performance of AI in predicting mortality in COVID-19 patients. The overall pooled AUROC for identifying sepsis patients was 0.93 [0.90, 0.95]. Additionally, the sensitivity, specificity, PLR, NLR, and diagnostic odds ratio were 0.84 [0.78, 0.89], 0.88 [0.84, 0.91], 6.9 [5.0, 9.5], 0.18 [0.13, 0.26], and 38 [22, 65], respectively (Figure S1-S4).  Training set  In the training set, a total of 14 models from 5 studies evaluated the performance of AI in predicting mortality in COVID-19 patients. The overall pooled AUROC for identifying sepsis patients was 0.98 [0.96, 0.99]. Additionally, the sensitivity, specificity, PLR, NLR, and diagnostic odds ratio were 0.93 [0.87, 0.96], 0.94 [0.87, 0.97], 15.08 [6.89, 33.01], 0.07 [0.04, 0.14], and 202.41 [49.05, 835.20], respectively (Figure S5-S8). | 8-9 |
| DISCUSSION | | |  |
| Strengths and limitations | 9 | First, the number of studies we included is relatively limited. Due to the lack of relevant articles on artificial intelligence models based on imaging features, we did not include them in the analysis. We hope that more studies will be conducted in the future. Ability to develop and validate models with imaging features. Second, there were as many as 25 AI models in our included articles, which we believe may be a major source of heterogeneity. Finally, in our included literature, baseline variables (e.g., demographic characteristics, vital signs, comorbidities, laboratory tests) included in each model differed to some extent, which may also be a source of some of the heterogeneity | 9 |
| Interpretation | 10 | Compared with traditional COVID-19 mortality screening tools, the artificial intelligence model has high accuracy in predicting the mortality of COVID-19 patients, better prediction performance, and higher prognostic value. Among them, KNN, SVM, RF, ANN, XGBoost and other models have higher accuracy. | 10 |
| OTHER | | |  |
| Funding | 11 | This work was supported by the Technology Department and Natural Science Foundation for Distinguished Young Scholars of Heilongjiang Province (JQ2021H003). | 15 |
| Registration | 12 | Prospero：CRD42022315158 |  |

*Adapted From:*  McInnes MDF, Moher D, Thombs BD, McGrath TA, Bossuyt PM, The PRISMA-DTA Group (2018). Preferred Reporting Items for a Systematic Review and Meta-analysis of Diagnostic Test Accuracy Studies: The PRISMA-DTA Statement. JAMA. 2018 Jan 23;319(4):388-396. doi: 10.1001/jama.2017.19163.

For more information, visit: **www.prisma-statement.org**.

Page 1 of 1
