# Supplementary material for: The accuracy of artificial intelligence in predicting COVID-19 patient mortality: a systematic review and meta-analysis
Source: BMC Med Inform Decis Mak. 2023 Aug 9;23:155. doi: 10.1186/s12911-023-02256-7 (PMC10410953; doi:10.1186/s12911-023-02256-7)
Supplement: Supplementary file 3 — Supplementary Material 3：Detailed retrieval strategy [file 12911_2023_2256_MOESM3_ESM.docx]

#### **Detailed retrieval strategy**

| ****Database**** | **Pubmed** |
| --- | --- |
| ****Website**** | **https://pubmed.ncbi.nlm.nih.gov** |
| ****Time**** | **database building - 2022.10.21** |
| ****Results**** | **570** |
| ****Search details**** | **Search: ((((Mortality[Title/Abstract]) OR ("Mortality"[Mesh])) AND (("Diagnosis"[Mesh]) OR (diagnosis[Title/Abstract]))) AND ((((((((((((((Artificial Intelligence[Title/Abstract]) OR (Machine Intelligence[Title/Abstract])) OR (ai[Title/Abstract])) OR (computer-aided diagnosis[Title/Abstract])) OR (Computational Intelligence[Title/Abstract])) OR (Computer Reasoning[Title/Abstract])) OR (deep learning[Title/Abstract])) OR (deep learning[Title/Abstract])) OR (support vector machine[Title/Abstract])) OR (random forest[Title/Abstract])) OR (decision tree[Title/Abstract])) OR (computed tomography[Title/Abstract])) OR (Supervised Machine Learning[Title/Abstract])) OR ((("Artificial Intelligence"[Mesh]) OR "Deep Learning"[Mesh]) OR "Machine Learning"[Mesh]))) AND (((covid-19[Title/Abstract]) OR (sars-cov-2[Title/Abstract])) OR (("COVID-19"[Mesh]) OR "SARS-CoV-2"[Mesh]))** |
| ****Database**** | **Embase** |
| ****Website**** | **https://www.embase.com** |
| ****Time**** | **database building - 2022.10.21** |
| ****Results**** | **1252** |
| ****Search details**** | **SourcesEmbase, MEDLINE, Preprints**  **Query('artificial intelligence'/exp OR 'machine learning'/exp OR 'deep learning'/exp OR 'artificial intelligence':ab,ti OR 'machine intelligence':ab,ti OR 'machine learning':ab,ti OR 'ai':ab,ti OR 'computer-aided diagnosis':ab,ti OR 'computational intelligence':ab,ti OR 'computer reasoning':ab,ti OR 'deep learning':ab,ti OR 'support vector machine':ab,ti OR 'random forest':ab,ti OR 'decision tree':ab,ti OR 'computed tomography':ab,ti OR 'supervised machine learning':ab,ti) AND ('coronavirus disease 2019'/exp OR 'coronavirus disease 2019':ab,ti OR 'covid-19':ab,ti OR 'sars-cov-2':ab,ti) AND ('diagnosis'/exp OR 'diagnosis':ab,ti) AND ('mortality'/exp OR 'mortality':ab,ti)** |
| ****Database**** | **Web of science** |
| ****Website**** | **http://www.webofscience.com** |
| ****Time**** | **database building - 2022.10.21** |
| ****Results**** | **293** |
| ****Search details**** | **#1 ((((TS=(artificial intelligence)) OR TS=(machine learning)) OR TS=(deep learning)) OR TS=(artificial intelligence)) OR TS=(machine intelligence)**  **#2 TS=(coronavirus disease 2019)  or TS=(covid-19)  or TS= (sars-cov-2)**  **#3 TS=(diagnosis)**  **#1 and #2 and #3** |
| ****Database**** | **CNKI (Chinese database)** |
| ****Website**** | **https://www.cnki.net** |
| ****Time**** | **database building - 2022.10.21** |
| ****Results**** | **7** |
| ****Search detail**** | **(Theme =AI + machine learning + AI + Deep learning + decision tree + Random forest) AND (theme =SARS-CoV-2 + COVID-19) AND (theme = mortality)** |
| ****Database**** | **Wanfang (Chinese database)** |
| ****Website**** | **https://www.wanfangdata.com.cn/index.html** |
| ****Time**** | **database building - 2022.10.21** |
| ****Results**** | **62** |
| ****Search details**** | (Theme =AI + machine learning + AI + Deep learning + decision tree + Random forest) AND (theme =SARS-CoV-2 + COVID-19) AND (theme = mortality) |
| ****Database**** | **China Biomedical Literature Database (Chinese database)** |
| ****Website**** | **http://www.sinomed.ac.cn/index.jsp** |
| ****Time**** | **database building - 2022.10.21** |
| ****Results**** | **7** |
| ****Search details**** | (Theme =AI + machine learning + AI + Deep learning + decision tree + Random forest) AND (theme =SARS-CoV-2 + COVID-19) AND (theme = mortality) |
| ****Database**** | **VIP Database (Chinese database)** |
| ****Website**** | **http://qikan.cqvip.com** |
| ****Time**** | **database building - 2022.10.21** |
| ****Results**** | **3** |
| ****Search details**** | (Theme =AI + machine learning + AI + Deep learning + decision tree + Random forest) AND (theme =SARS-CoV-2 + COVID-19) AND (theme = mortality) |
