# Supplementary material for: The accuracy of artificial intelligence in predicting COVID-19 patient mortality: a systematic review and meta-analysis
Source: BMC Med Inform Decis Mak. 2023 Aug 9;23:155. doi: 10.1186/s12911-023-02256-7 (PMC10410953; doi:10.1186/s12911-023-02256-7)
Supplement: Supplementary file 4 — Supplementary Material 4：AI Model Detailed Predictors [file 12911_2023_2256_MOESM4_ESM.docx]

**AI Model Detailed Predictors**

| **Study ID** | **Predictors** |
| --- | --- |
| Ashis Kumar Das^[10]^ | Sex,age,province,and exposure. |
| Limin Yu^[11]^ | Vented,Respiration,BMI,LOS,Race,Pulse,ICU adm,dBP,Temp,Pressors,tACdur,Steroid dur,Heart,DM,Cancer,Steroid,tAC,HTN,Sex. |
| Fabiana Tezza^[12]^ | Age,Creatinine,AST,Oxygen saturation,Lymphocytes,Platelets,Hemoglobin,Quick SOFA 2,ALT,Neutrophils,Dementia,Smoking,LMWH,Quick SOFA 1,Chronic Kidney Disease,SHPT,Current cancer,Gender,Dyslipidemia,Diabetes,Mellitus,Cerebrovascular disease,COPD,Ischemic heart disease,Prior cancer,Quick SOFA 3,Obesity. |
| Alejandro Santos-Lozano^[13]^ | more advanced age,higher levels of serum lactate dehydrogenase(LDH)and lower levels of glomerular filtration rate(GFR),albumin and hemoglobin |
| Prathamesh Parchure^[14]^ | Age,Blood urea nitrogen,Respiratory rate,T wave axis,Serum chloride,Platelet count,Serum creatinine,Anion gap,Atrial rate,Corrected WBC count,Red blood cell count,Partial pressure of,carbon dioxide in arterial blood(PaC02),C-reactive protein,erum sodium,Diastolic blood pressure,Hemoglobin,Lenath of stay,PR interval. |
| Rita Murri^[15]^ | Age,Sp02<=94.3%,Platelets(10^9/1),Neutrophils(10^9/1),SpO2 94.4-97.0%,Sodium>141 mmol/l,Hemoglobin(g/dI),C-reactive protein(mg/l),Blood urea nitrogen(mg/dI),Sodium<136 mmol/l. |
| Xiaoran Li^[16]^ | Age,LDH,Procalcitonin,Troponin,CRP,Sp02,Heart Failure history,RR,Lymphocytes,Ferritin,COPD history,D-dimer,ALT,Coronary heart disease history,Systolic BP,HRCough. |
| Sujoy Kar^[17]^ | Age>60 years,Gender(male),Fever,Cough,Respiratory distress,Weakness,Diabetes,Hypertension,Chronic kidney disease,Chronic liver disease,Heart disease,ROROxygen saturation(SpO2),WBC count,Lymphocytes%,IN(prothrombin time),Creatinine,Albumin,AST(liver enzyme),Lactate dehydrogenase,Ferritin,C-reactive protein,Red cell distribution width. |
| Adam L. Booth^[18]^ | C-Reactive Protein,Calcium,Lactic Acid,Blood Urea Nitrogen,Albumin. |
| Emirena Garrafa^[19]^ | age,ldh,d-dimer,neutr/lymph,neutrophils%,fibrinogen,crp,monocytes%,lymphocytes%,ferritin std,brescia chest-xray,monocytes,lymphocytes,eosinophils%,neutrophils,wbc,eosinophils,basophils%,basophils. |
| Mohammad M. Banoei^[20]^ | Coronary artery disease,diabetes,Altered Mental Status,age>65,and dement. |
| Hoon Ko^[21]^ | Lymphocyte,Neutrophils,Albumin,Lactate dehydrogenase,Neutrophil count Hypersensitive-reactive protein,Prothrombin activity,Calcium,Urea,Estimated glomerular filtration rate,Monocytes,Globulin,Eosinophils,Glucose,Red blood cell distribution width(RDW),HC03-(bicarbonate),RDW standard deviation(RDW-SD),Platelet count,Mean platelet volume Platelet large cell ratio,Prothrombin time,Total protein,Platelet distribution width,Aspartate aminotransferase,Thrombocytosis,Eosinophil count,Alkaline phosphatase,International standard ratio. |
| Ju-Kuo Lin^[22]^ | Age,Gender,Lymphocyte,Neutrophils,Albumin,LDH,Neutrophils count,hs-CRP,Prothrombin activity,Calcium,Urea,eGFR,Monocytes,Globulin,Eosinophils,Glucose,RDW,HC03-,RDW,Platelet count,Mean platelet volume,Platelet large cell ratio,PT(Prothrombin time),Total protein,PLT distribution width,AST(Aspartate aminotransferase),Thrombocytocrit,Eosinophil count,ALP(Alkaline phosphatase),IN(International standard ratio). |
| Ahmed Abdulaal^[23]^ | Altered mentation,Dyspnea,Age,Collapse,Gender,Cough,Respiratory pathology,Hypertension,Fever,Chronic kidney disease,Ischemic heartdiseaseCerebrovascular event,Myalgia,Smoking history,Cardiac failure,Days of symptoms,Obesity,Diarrhea or vomiting,Anosmia and/or ageusia,Liver,cirrhosis,Diabetes,Abdominal pain. |
| Fatemeh Moghaddam-Tabrizi^[24]^ | Age,Pulmonary symptoms,Ventilator,brain symptoms,Nasal Airway,Job,Gastrointestinal symptoms,Brain disease history,Heart disease history,Heart symptoms,Chronic Kidney disease history,Psychological symptoms,Dyspnea symptom,History of drug use,Hypertension history,Resident. |
| Abdulrhman Fahad Aljouie^[25]^ | / |
| Maleeha Naseem^[26]^ | / |
| Khadijeh Moulaei^[27]^ | Dyspnea,Underlying diseases,Headache,Weakness and lethargy,Body pain,Fatigue,Sore throat,Age,Dry cough,Diarrhea,Pain or,pressure in the chest,High fever,Loss of sense of smell and,taste,Nausea and Vomiting,Anorexia,Gender. |
| Logan Ryan^[28]^ | SysABP,Platelets,Sp02,DiasABP,Lactate,RespRate,SysABP,Creatinine,Temp,RespRate. |
| Kenji Ikemura^[29]^ | Age,Gender,Race,Albumin,Diastolic BP,Systolic BP,Cr,D-Dimer,eGRF,Eosinophil,Ferritin,Fibrinogen,Hgb,INR,Lymphocyte,Neutrophil,N/L Ratio,Platelet,Protein,Pulse,Pulse Ox,RR,Temperature,WBC,ALT,AST,BUN,Calcium,Chloride,CRP,Interleukin 6,LDH,MCV,Monocyte,MPV,Procalcitonin,RDW,Troponin,PTT,BMI,Glucose,Direct Bilirubin,Total Bilirubin,Creatine Kinase,Pro BNP,Potassium,Charlson Score,Ct value. |
| Chi Peng^[30]^ | Age,Male,RR,Pulse,SBP,DBP,Cough,Fatigue,Fever,Sputum,Gasp,WBC,Lymphocyte,Monocyte,Neutrophil,Eosinophil, Basophil,RBC, Hemoglobin, Hematocrit,MCV,MCH, Platelet, MPV, hs-CRP,ALT,AST,ALP,TP, Albumin, Creatinine, TBil, DBil, CO_2,_ TBA,BUN,UA, Na＋, K＋, Ca2＋, Cl－, CK-MB, Hypertension, Diabetes,CHD,COPD, Kidney disease, Cancer. |
| Nicolás Munera^[31]^ | Age, FiO2, Systolic blood pressure, Diastolic blood pressure, SO2 , Glasgow Coma Scale, Sex, Dyspnoea, Obesity, Vomiting/nausea, Abdominal pain, Chronic kidney disease, Conjunctivitis, Arterial hypertension, Skin ulcers, Diabetes mellitus. |
| Hongbing Peng^[32]^ | Age, Gender, Imput case (close contact with Wuhan), Frequent breathing (cpm) median, Blood pressure on admission, Systolic pressure, Diastolic pressure, Temperature, Dyspnea, Cough, Headache, Fatigue, Muscle soreness, Gastrointestinal symptoms, Incubation period (days) median, Length of hospital stay (days) median, Comorbidities, Respiratory system disease, Cardiovascular diseases, Endocrine system disease and metabolic related diseases, Hypertension, Malignant tumour, Digestive disease, White cell count, Neutrophil count, Lymphocyte count, NLR, Platelet count, Haemoglobin, D-dimer, Albumin, Myoglobin, C-reactive protein, Creatine kinase, Creatine kinase MB, Lactate dehydrogenase, Blood urea nitrogen, Creatinine, Procalcitonin, Blood Glucose, Total bilirubin, Direct bilirubin, Semi-quantitative chest CT Score. |

**brief introduction to each AI model**

1. KNN: One of the simplest algorithms in classification algorithm, its core idea is that if most of the k samples nearest to a sample belong to a certain category, the sample also belongs to this category, and has the characteristics of samples on this category.

Advantage:

1. It can be used for nonlinear classification;
2. The training time complexity is lower than that of algorithms like support vector machine;
3. Compared with algorithms like Naive Bayes, it has no assumptions on data, high accuracy and is insensitive to outliers;

Disadvantages:

1. Large amount of calculation, especially when the number of features is very large;
2. When the sample is unbalanced, the prediction accuracy of rare categories is low;
3. Compared with the decision tree model, the KNN model is not strong in interpretability;
4. SVM:It is a supervised learning method, which can be widely used in statistical classification and regression analysis. The vector is mapped to a higher dimensional space in which a maximally spaced hyperplane is established. There are two hyperplanes parallel to each other on either side of the hyperplane separating the data. The separation of the hyperplanes maximizes the distance between the two parallel hyperplanes. It is assumed that the larger the distance or gap between parallel hyperplanes, the smaller the total error of the classifier.

Advantages:

1. Good classification effect;
2. The kernel function can be used to map to higher dimensional space;
3. Kernel function can be used to solve nonlinear classification;

Disadvantages:

1. SVM algorithm is difficult to implement for large-scale training samples;
2. Sensitive to missing data and the selection of parameters and kernel functions;
3. RF: Random Forest is a classifier that contains multiple decision trees, and its output category is determined by the mode of the category output by the individual tree. Random Forest (RF) builds multiple decision trees and merges them together to obtain more accurate and stable predictions.

Advantages:

1. High accuracy;
2. Can handle a large number of input variables and evaluate the importance of variables;
3. The introduction of randomness makes random forest not easy to overfit;

Disadvantages:

1. When the number of decision trees in random forest is large, the space and time required for training will be large;
2. ANN:Artificial neural network abstracts the brain neuron network from the perspective of information processing, builds some simple models, and forms different networks according to different connection modes.

Advantages:

1. High accuracy;
2. Strong parallel distribution processing ability;
3. High fault tolerance to noise;

Disadvantages:

1. A large number of initial parameters are required;
2. XGBoost:The integrated machine learning algorithm based on decision tree uses gradient lifting framework. Ann tends to outperform all other algorithms or frameworks in prediction problems involving unstructured data (images, text, etc.). However, when it comes to small and medium-sized structured/tabular data, decision tree-based algorithms are considered to be the best in their class at present.

Advantages:

1. XGBoost not only uses the first derivative, but also uses the second derivative, loss is more accurate, but also can customize the loss ;
2. Considering that the training data are sparse values, the default direction of branches can be specified for missing values or specified values, which can greatly improve the efficiency of the algorithm;
3. LR:Regression analysis is a predictive modeling technique that studies the relationship between independent variables and dependent variables. LR (Linear regression) is the most basic regression algorithm. The line (surface) model is used to fit the existing relatively linear data with less loss, and the fitted model can better predict the data

Advantages:

1. The possibility of classification is modeled directly without prior assumption of data distribution, which avoids the problems caused by inaccurate assumption distribution;
2. Not only the category can be predicted, but also the approximate probability prediction can be obtained;

Disadvantages:

1. It is easy to underfit and the classification accuracy is not high;
2. The effect is not good when the data features are missing or the feature space is large;
3. DNN:MLP (Multi-Layer Perceptron), which is extended from PLA (Perceptron Learning Algorithm), is a neural network that adds one or several hidden layers on the basis of single-layer neural network. The hidden layer is between the input layer and the output layer.

Advantages:

1. The model structure is very flexible, with high degree of freedom and easy to construct;

Disadvantages:

1. The analytical performance is poor, and the training consumes more time and space resources;
2. GBM:Ensemble is a kind of ensemble learning method. Ensemble learning method (also known as combinatorial learning method) is a machine learning method that uses a series of learners to learn, and uses certain rules to integrate each learning result, so as to obtain better learning effect than a single learner. Boosting is a kind of boosting algorithm, which linearly combines the base learners with different weights, so that the best performers can be reused. Boosting comes in various forms depending on the base learner, the loss function, and the optimization method. The GBM algorithm calculates the pseudo-residuals according to the initial model, and then builds a basis learner to interpret the pseudo-residuals, which reduces the residuals in the gradient direction. Then the base learner is multiplied by the weight coefficient (learning rate) and the original model is linearly combined to form a new model. In this way, we can find a model that minimizes the expectation of the loss function.

Advantages:

1. Inherited the advantages of single decision tree, but abandoned its disadvantages;
2. Able to handle missing data;
3. Insensitive to noisy data;
4. Can fit complex nonlinear relationships;

Disadvantages:

1. Overfitting may occur;
2. Set too many parameters;
3. Weak anti-interference ability;
4. Able to fit complex nonlinear relationships;
5. DT: Decision tree is based on the known probability of occurrence of various situations, through the formation of decision tree to obtain the probability that the expected value of net present value is greater than or equal to zero, evaluation of project risk, judge its feasibility of decision analysis method, is a visual use of probability analysis of a graphical method.

Advantages:

1. Decision tree is easy to understand and implement, people do not need to know a lot of background knowledge in the learning process, this is also its characteristics can directly reflect the data, as long as the interpretation of the ability to understand the meaning of the decision tree;

Disadvantages:

1. The generation of decision trees is unstable, and small data changes may result in different generated decision trees;

| **Model** | | **Software** | **Website, References** |
| --- | --- | --- | --- |
| KNN | K-Nearest Neighbor | R,Python | 1. <https://scikit-learn.org/stable/modules/neighbors.html> 2. <http://www.tutorialspoint.com/machine_learning_with_python/machine_learning_with_python_knn_algorithm_finding_nearest_neighbors.htm> |
| SVM | Support Vector Machine | Python,R | 1. Lundberg SM,Lee S-I.A unified approach to interpreting model predictions.In:Advances in neural information processing A.L.Booth et al.systems.2017.https://papers.nips.cc/paper/7062-a-unified-approach-to-interpreting-model-predictions.pdf. 2. http://www.saedsayad.com/support_vector_machine.htm |
| RF | Random forests | R, Matlab | 1. <https://www.stat.berkeley.edu/~breiman/RandomForests/cc_home.htm#inter> 2. https://www.ibm.com/cloud/learn/random-forest |
| ANN | Artificial Neural Network | Python,R | 1. <https://developer.nvidia.com/discover/artificial-neural-network> 2. https://brilliant.org/wiki/artificial-neural-network/ |
| XGBoost | eXtreme Gradient Boosting | Python, R, Java, | 1. <https://github.com/dmlc/xgboost/> |
| LR | Logistic Regression | Matlab,R | 1. <https://scikit-learn.org/stable/modules/generated/sklearn.linear_model.LogisticRegression.html#> |
| DNN | Deep neural network | Boruta,R | 1. Kursa MB, Rudnicki WR. 2010. Feature selection with the Boruta package. Journal of Statistical Software 36(11):1–13 2. <https://www.classcreator.com/Kaneohe-Hawaii-None-1900/class_index.cfm> 3. Cai, H., Lin, J., Han, S., 2022. Chapter 4 - Efficient methods for deep learning , Advanced Methods and Deep Learning in Computer Vision. Academic Press, pp. 159-190. |
| GBM | Gradient Boosting Machine | R | 1. <https://www.analyticsvidhya.com/blog/2016/02/complete-guide-parameter-tuning-gradient-boosting-gbm-python/> 2. Natekin A, Knoll A. Gradient boosting machines, a tutorial. Front Neurorobot. 2013 Dec 4;7:21. doi: 10.3389/fnbot.2013.00021. PMID: 24409142; PMCID: PMC3885826. |
| DT | Decision tree | Matlab,R | 1. <https://scikit-learn.org/stable/modules/tree.html> 2. Breiman, L., J. H. Friedman, R. A. Olshen, and C. J. Stone. Classification and Regression Trees. Boca Raton, FL: Chapman & Hall, 1984 |
