# Supplementary material for: The accuracy of artificial intelligence in predicting COVID-19 patient mortality: a systematic review and meta-analysis
Source: BMC Med Inform Decis Mak. 2023 Aug 9;23:155. doi: 10.1186/s12911-023-02256-7 (PMC10410953; doi:10.1186/s12911-023-02256-7)
Supplement: Supplementary file 5 — Supplementary Material 5：Table S1-S2, Figure S1-S9 [file 12911_2023_2256_MOESM5_ESM.docx]

**Table s1 Validation set basic information**

| Author | Country | Model | Alive | Dead | TP | FP | FN | TN | SEN | SPE | AUC |
| --- | --- | --- | --- | --- | --- | --- | --- | --- | --- | --- | --- |
| Ashis Kumar Das | Korea | LR | 690 | 15 | 10 | 22 | 5 | 668 | 0.69 | 0.97 | 0.83 |
|  |  | SVM | 690 | 15 | 10 | 31 | 5 | 659 | 0.69 | 0.96 | 0.82 |
|  |  | KNN | 690 | 15 | 5 | 13 | 10 | 677 | 0.31 | 0.98 | 0.64 |
|  |  | RF | 690 | 15 | 9 | 28 | 6 | 662 | 0.62 | 0.97 | 0.78 |
|  |  | XGBoost | 690 | 15 | 9 | 28 | 6 | 662 | 0.62 | 0.96 | 0.78 |
| Limin Yu | Mexico | Cat boost | 98 | 105 | 79 | 21 | 19 | 84 | 0.82 | 0.78 | 0.85 |
| Fabiana Tezza | Italy | RPART | 266 | 75 | 33 | 17 | 42 | 249 | 0.44 | 0.94 | 0.64 |
|  |  | SVM | 266 | 75 | 68 | 111 | 7 | 155 | 0.91 | 0.58 | 0.78 |
|  |  | GBM | 266 | 75 | 64 | 90 | 11 | 176 | 0.85 | 0.66 | 0.81 |
|  |  | RF | 266 | 75 | 59 | 60 | 16 | 206 | 0.79 | 0.77 | 0.84 |
| Alejandro Santos-Lozano | Spain | ANN | 1090 | 279 | 265 | 425 | 14 | 665 | 0.95 | 0.61 | 0.91 |
| Prathamesh Parchure | USA | RF | 267 | 56 | 49 | 105 | 7 | 162 | 0.88 | 0.61 | 0.86 |
| Rita Murri | Italy | LR | 1131 | 332 | 270 | 396 | 62 | 735 | 0.81 | 0.65 | 0.82 |
| Xiaoran Li | USA | DNN | 86 | 16 | 12 | 11 | 4 | 75 | 0.75 | 0.87 | 0.84 |
| Sujoy Kar | India | XGBoost | 896 | 81 | 47 | 34 | 30 | 866 | 0.61 | 0.96 | 0.93 |
| Adam L. Booth | USA | SVM | 69 | 11 | 10 | 6 | 1 | 63 | 0.91 | 0.91 | 0.93 |
| Emirena Garrafa | Italy | RF | 594 | 82 | 60 | 160 | 22 | 434 | 0.73 | 0.73 | 0.78 |
|  |  | GBM | 594 | 82 | 66 | 148 | 16 | 446 | 0.80 | 0.75 | 0.84 |
|  |  | LR | 594 | 82 | 71 | 440 | 11 | 154 | 0.87 | 0.26 | 0.52 |
| Mohammad M. Banoei | USA | SIMPLS | 68 | 10 | 8 | 7 | 2 | 61 | 0.75 | 0.90 | 0.91 |
| Hoon Ko | Korea | XGBoost | 104 | 2 | 2 | 24 | 0 | 80 | 1.00 | 0.77 | / |
|  |  | ADABoost | 104 | 2 | 2 | 23 | 0 | 81 | 1.00 | 0.78 | / |
|  |  | RF | 104 | 2 | 2 | 17 | 0 | 87 | 1.00 | 0.84 | / |
|  |  | DNN | 104 | 2 | 1 | 9 | 1 | 95 | 0.50 | 0.91 | / |
|  |  | DNN + XGBoost | 104 | 2 | 2 | 24 | 0 | 80 | 1.00 | 0.77 | / |
|  |  | DNN + AdaBoost | 104 | 2 | 1 | 8 | 1 | 96 | 0.50 | 0.92 | / |
|  |  | DNN + RF | 104 | 2 | 2 | 9 | 0 | 95 | 1.00 | 0.91 | / |
| Ju-Kuo Lin | China | CNN | 104 | 2 | 2 | 56 | 0 | 48 | 1.00 | 0.46 | / |
|  |  | ANN | 104 | 2 | 2 | 42 | 0 | 62 | 1.00 | 0.60 | / |
|  |  | Forest(J48) | 104 | 2 | 1 | 23 | 1 | 81 | 0.50 | 0.78 | / |
|  |  | RF | 104 | 2 | 2 | 22 | 0 | 82 | 1.00 | 0.79 | / |
|  |  | Random tree | 104 | 2 | 2 | 16 | 0 | 88 | 1.00 | 0.85 | / |
|  |  | REPT tree | 104 | 2 | 2 | 31 | 0 | 73 | 1.00 | 0.73 | / |
|  |  | BAYESNET | 104 | 2 | 2 | 18 | 0 | 86 | 1.00 | 0.83 | / |
|  |  | Naïve Bayes | 104 | 2 | 2 | 18 | 0 | 86 | 1.00 | 0.83 | / |
|  |  | LOGISTIC | 104 | 2 | 2 | 29 | 0 | 75 | 0.72 | 0.73 | / |
|  |  | SMO | 101 | 2 | 1 | 10 | 1 | 91 | 0.50 | 0.90 | / |
| Ahmed Abdulaal | UK | ANN | 62 | 18 | 16 | 9 | 2 | 53 | 0.88 | 0.86 | 0.90 |
| Fatemeh Moghaddam-Tabrizi | Iran | RF | 316 | 85 | 80 | 14 | 5 | 302 | 0.95 | 0.96 | 0.83 |
| Abdulrhman Fahad Aljouie | Saudi Arabia | RF | 274 | 27 | 27 | 121 | 0 | 153 | 1.00 | 0.56 | 0.82 |
| Maleeha Naseem | Pakistan | DNN | 326 | 39 | 27 | 43 | 12 | 183 | 0.67 | 0.87 | 0.87 |
|  |  | Deep-FLAIM | 326 | 39 | 13 | 15 | 26 | 311 | 0.33 | 0.95 | 0.89 |
|  |  | RF | 326 | 39 | 11 | 20 | 28 | 306 | 0.29 | 0.94 | 0.70 |
|  |  | KNN | 326 | 39 | 16 | 56 | 23 | 270 | 0.42 | 0.83 | 0.67 |
|  |  | SVC-RBF | 326 | 39 | 29 | 115 | 10 | 211 | 0.76 | 0.64 | 0.81 |
|  |  | DT | 326 | 39 | 16 | 29 | 23 | 297 | 0.42 | 0.91 | 0.67 |
|  |  | ABC | 326 | 39 | 19 | 31 | 20 | 295 | 0.49 | 0.91 | 0.78 |
|  |  | QDA | 326 | 39 | 23 | 29 | 16 | 297 | 0.60 | 0.91 | 0.81 |
| Khadijeh Moulaei | Iran | DT | 650 | 200 | 196 | 17 | 4 | 633 | 0.98 | 0.97 | 0.98 |
|  |  | Multilayer perceptron | 650 | 200 | 191 | 8 | 9 | 642 | 0.95 | 0.99 | 0.99 |
|  |  | KNN1 | 650 | 200 | 191 | 14 | 9 | 636 | 0.95 | 0.98 | 0.99 |
|  |  | KNN2 | 650 | 200 | 196 | 0 | 4 | 650 | 0.98 | 1.00 | 0.98 |
|  |  | KNN3 | 650 | 200 | 190 | 31 | 10 | 619 | 0.95 | 0.95 | 0.99 |
|  |  | Random Forest | 650 | 200 | 197 | 1 | 3 | 649 | 0.98 | 0.99 | 1.00 |
|  |  | SVM | 650 | 200 | 196 | 26 | 4 | 624 | 0.98 | 0.96 | 0.97 |
| Logan Ryan | USA | XGBoost | 90 | 24 | 20 | 22 | 4 | 68 | 0.81 | 0.76 | 0.87 |
| Kenji Ikemura | USA | XGBoost（48） | 648 | 197 | 181 | 172 | 16 | 476 | 0.92 | 0.74 | / |
|  |  | XGBoost（10） | 648 | 197 | 165 | 106 | 32 | 542 | 0.84 | 0.84 | / |
| Chi Peng | China | ANN | 4704 | 100 | 81 | 216 | 19 | 4704 | 0.81 | 0.95 | 0.95 |
|  |  | NB | 4704 | 100 | 75 | 155 | 25 | 4704 | 0.75 | 0.97 | 0.96 |
|  |  | LR | 4704 | 100 | 75 | 216 | 25 | 4704 | 0.75 | 0.95 | 0.951 |
| Nicolás Munera | Colombia | RF | 1948 | 604 | 429 | 487 | 175 | 1948 | 0.71 | 0.75 | 0.81 |
| Hongbing Peng | China | LR | 60 | 2 | 1 | 15 | 1 | 60 | 0.55 | 0.75 | 0.68 |

**Table s2 Training set basic information**

| Author | Country | Model | Alive | Dead | TP | FP | FN | TN | SEN | SPE | AUC |
| --- | --- | --- | --- | --- | --- | --- | --- | --- | --- | --- | --- |
| Rita Murri | Italy | LR | 832 | 119 | 100 | 193 | 639 | 19 | 0.84 | 0.77 | 0.87 |
| Xiaoran Li | USA | DNN | 794 | 126 | 89 | 237 | 557 | 37 | 0.71 | 0.70 | 0.75 |
| Emirena Garrafa | Italy | RF | 888 | 888 | 817 | 62 | 826 | 71 | 0.92 | 0.93 | 0.97 |
|  |  | GBM | 888 | 888 | 755 | 204 | 684 | 133 | 0.85 | 0.77 | 0.88 |
|  |  | LR | 888 | 888 | 710 | 231 | 657 | 178 | 0.80 | 0.74 | 0.84 |
| Mohammad M. Banoei | USA | SIMPLS | 151 | 21 | 17 | 12 | 139 | 4 | 0.80 | 0.92 | 0.95 |
| Ju-Kuo Lin, MD | China | CNN | 195 | 166 | 154 | 14 | 181 | 12 | 0.93 | 0.93 | 0.91 |
|  |  | ANN | 195 | 166 | 158 | 4 | 191 | 8 | 0.95 | 0.98 | 0.96 |
|  |  | Forest(J48) | 195 | 166 | 164 | 6 | 189 | 2 | 0.99 | 0.97 | 0.97 |
|  |  | RF | 195 | 166 | 166 | 0 | 195 | 0 | 1.00 | 1.00 | 1.00 |
|  |  | Random tree | 195 | 166 | 166 | 0 | 195 | 0 | 1.00 | 1.00 | 1.00 |
|  |  | REPT tree | 195 | 166 | 153 | 14 | 181 | 13 | 0.92 | 0.93 | 0.91 |
|  |  | BAYESNET | 195 | 166 | 153 | 16 | 179 | 13 | 0.92 | 0.92 | 0.90 |
|  |  | Naïve Bayes | 195 | 166 | 133 | 16 | 179 | 33 | 0.80 | 0.92 | 0.90 |
|  |  | LOGISTIC | 195 | 166 | 154 | 16 | 179 | 12 | 0.93 | 0.92 | 0.91 |
|  |  | SMO | 195 | 166 | 144 | 14 | 181 | 22 | 0.87 | 0.93 | 0.88 |

**Figure s1 Forest plots of the pooled sensitivity and specificity for all AI models validation sets**


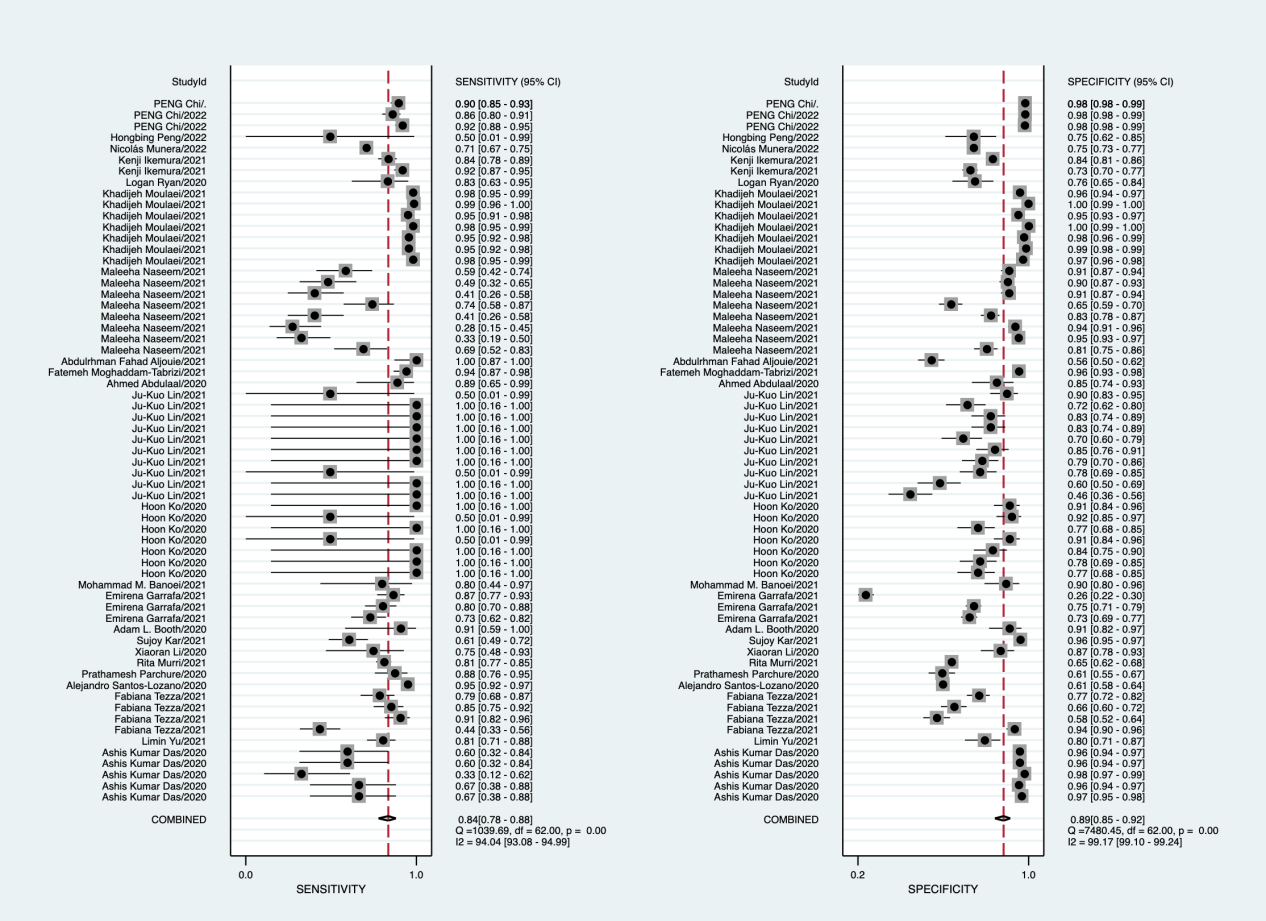


**Figure s2 Forest plot of the pooled positive LR and negative LR for all AI models validation sets**


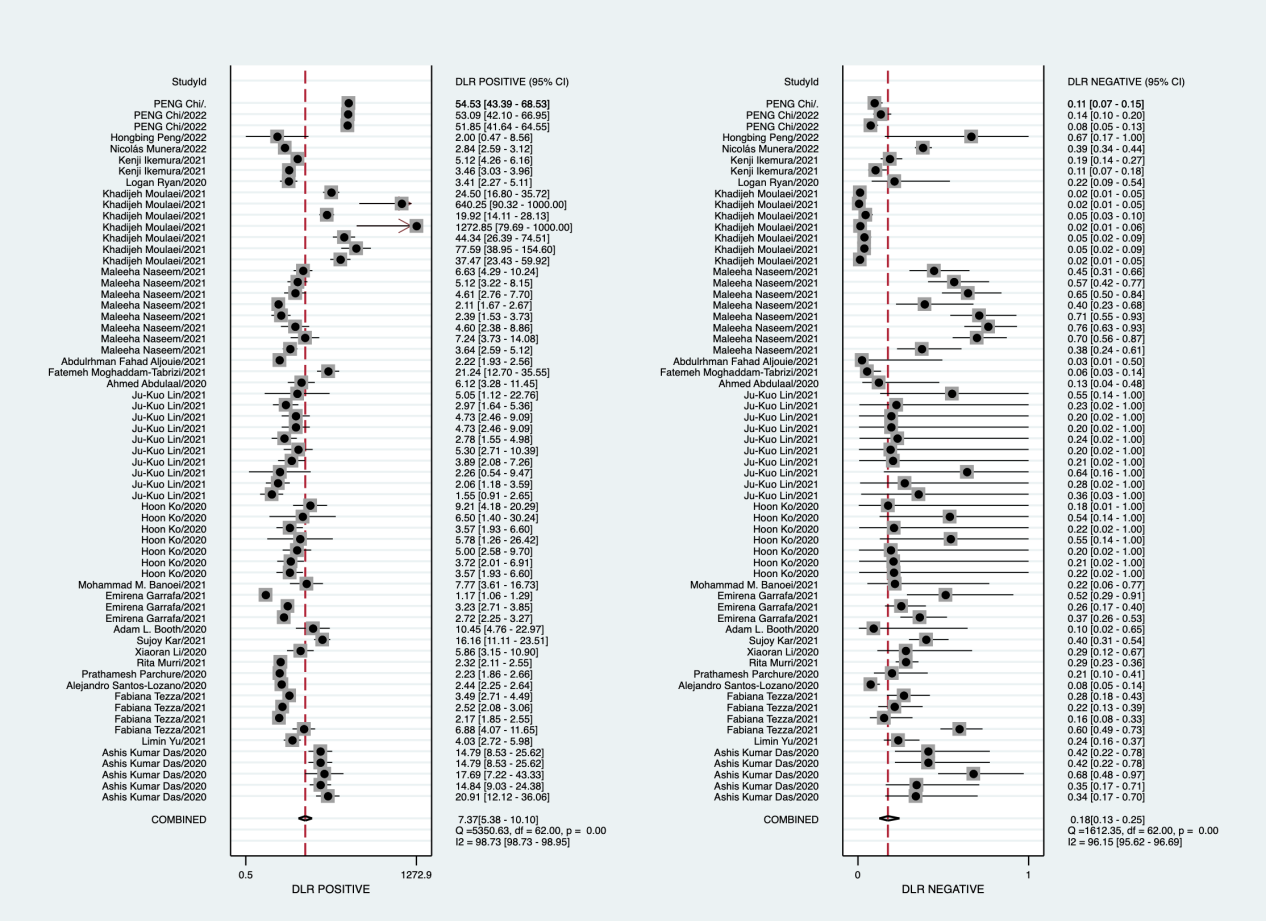


**Figure s3 Forest plot of the pooled diagnostic odds ratio for all AI models validation sets**


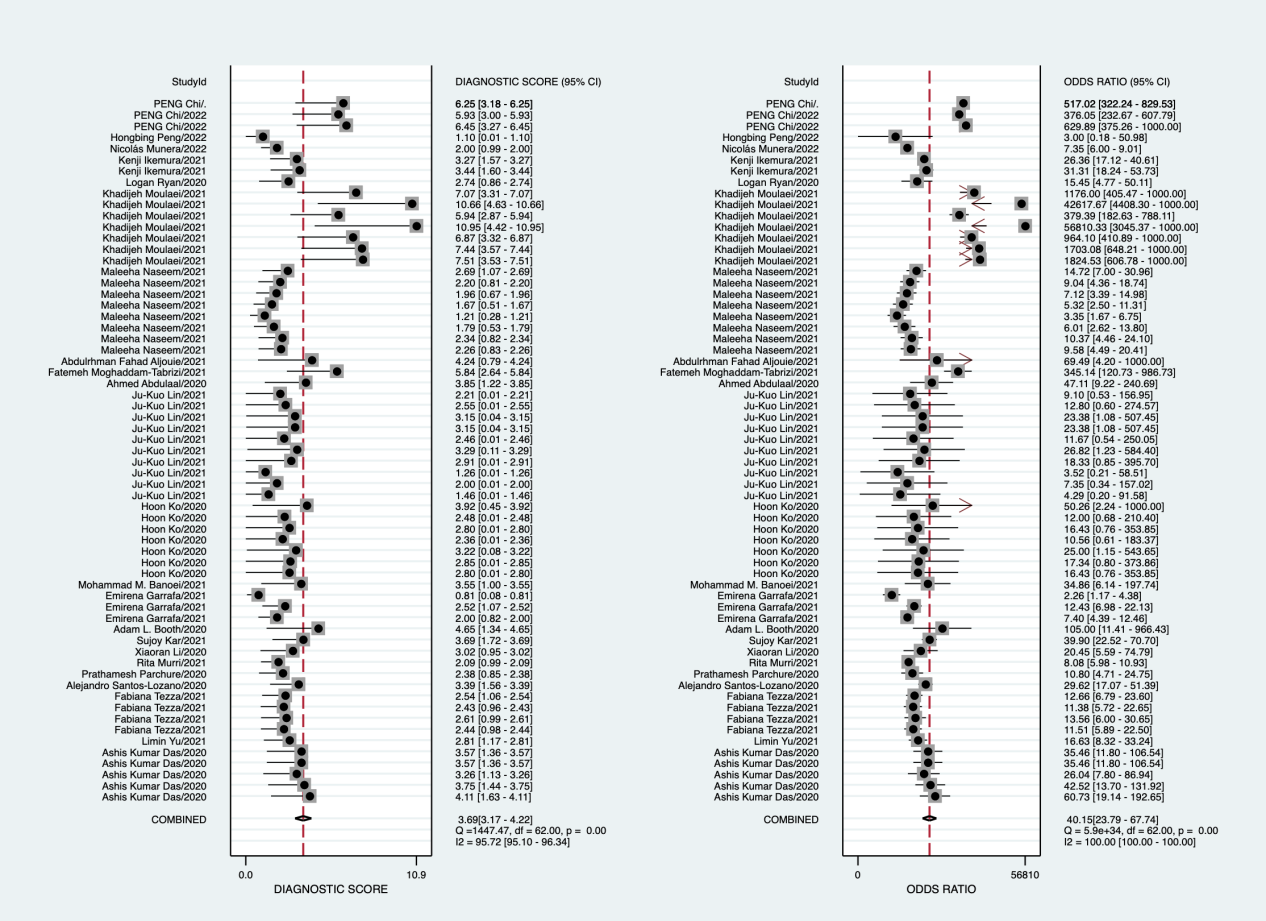


**Figure s4 SROC of AI for the diagnosis of COVID-19 patient mortality for all AI models validation sets**


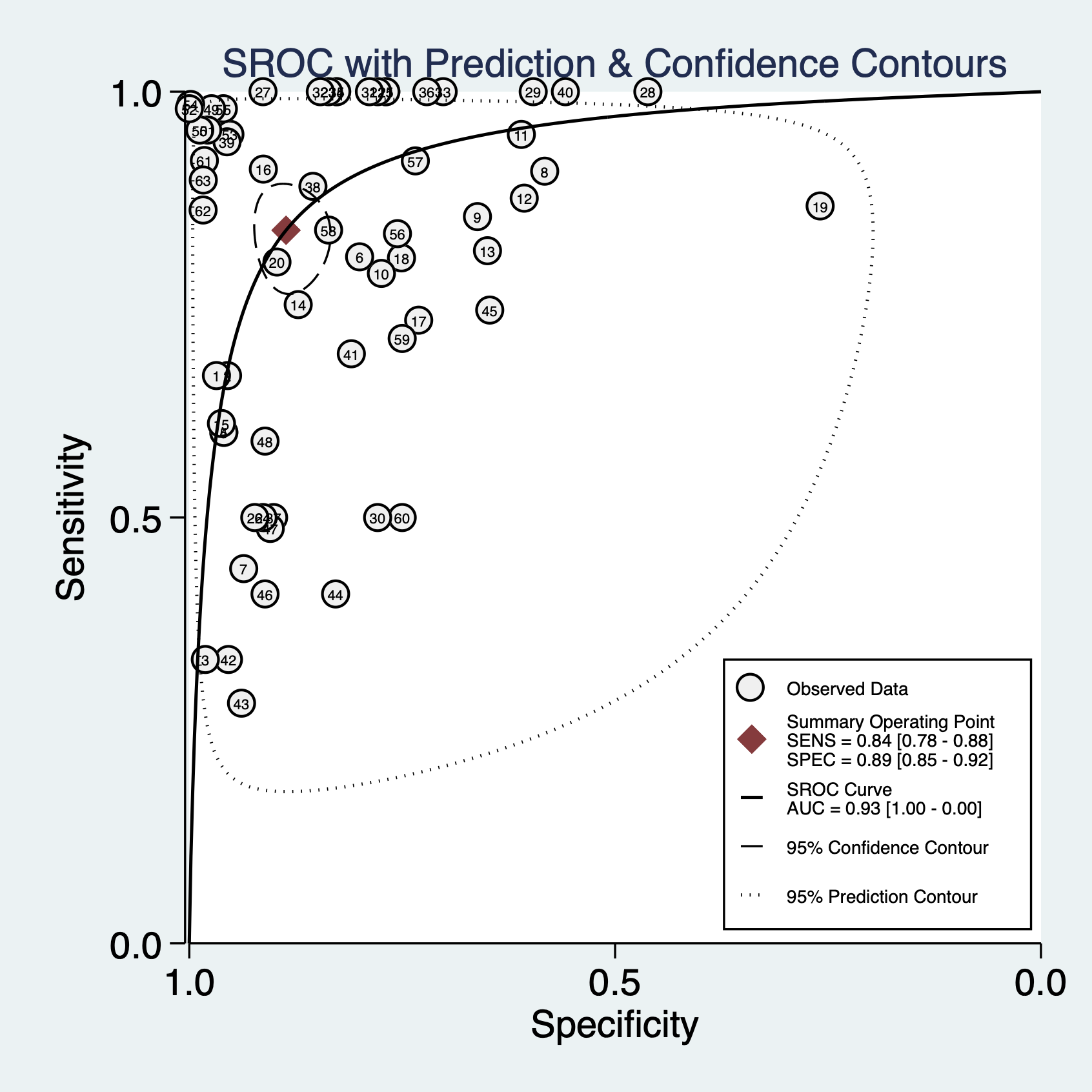


**Figure s5 Forest plots of the pooled sensitivity and specificity for all AI models training sets**


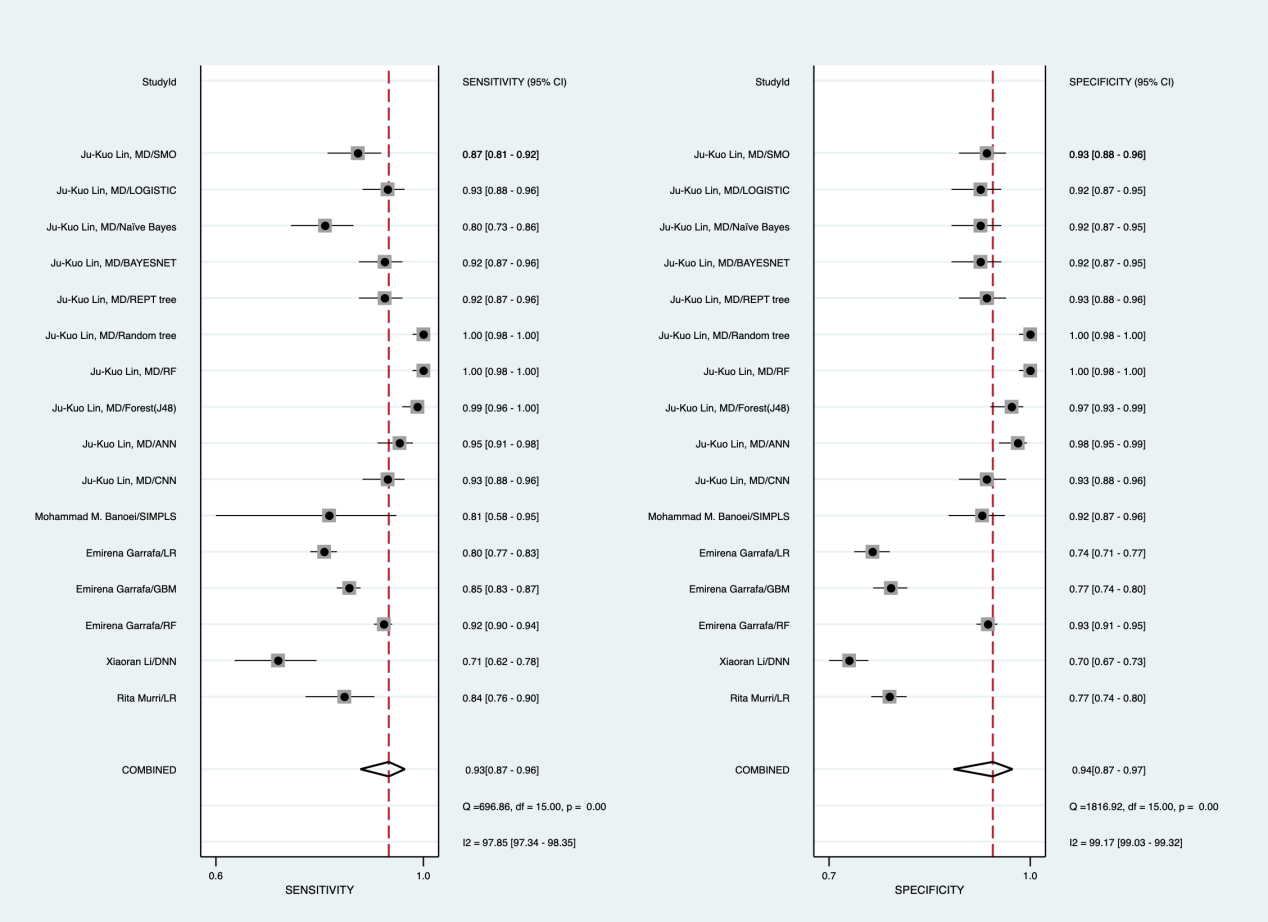


**Figure s6 Forest plot of the pooled positive LR and negative LR for all AI models training sets**


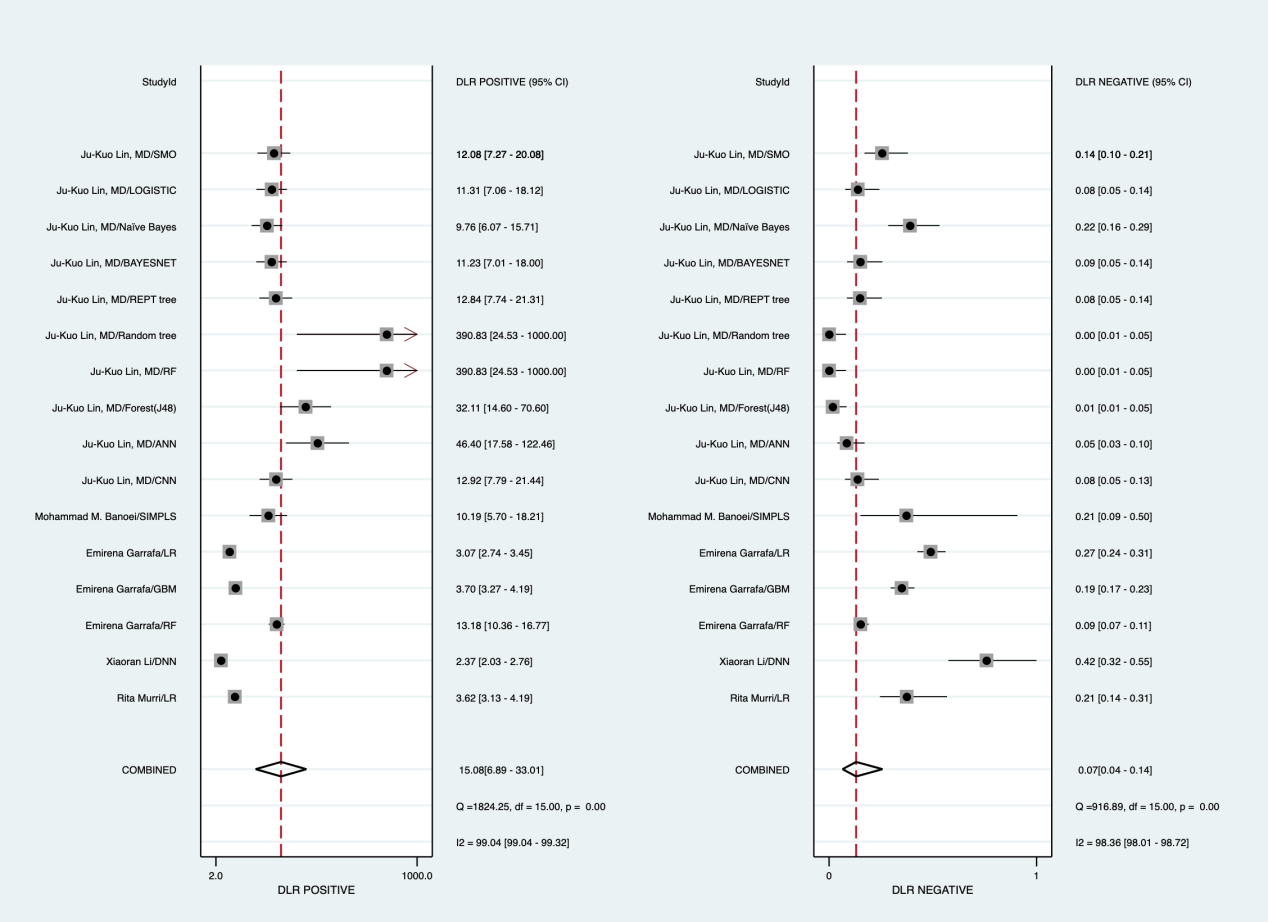


**Figure s7 Forest plot of the pooled diagnostic odds ratio for all AI models training sets**


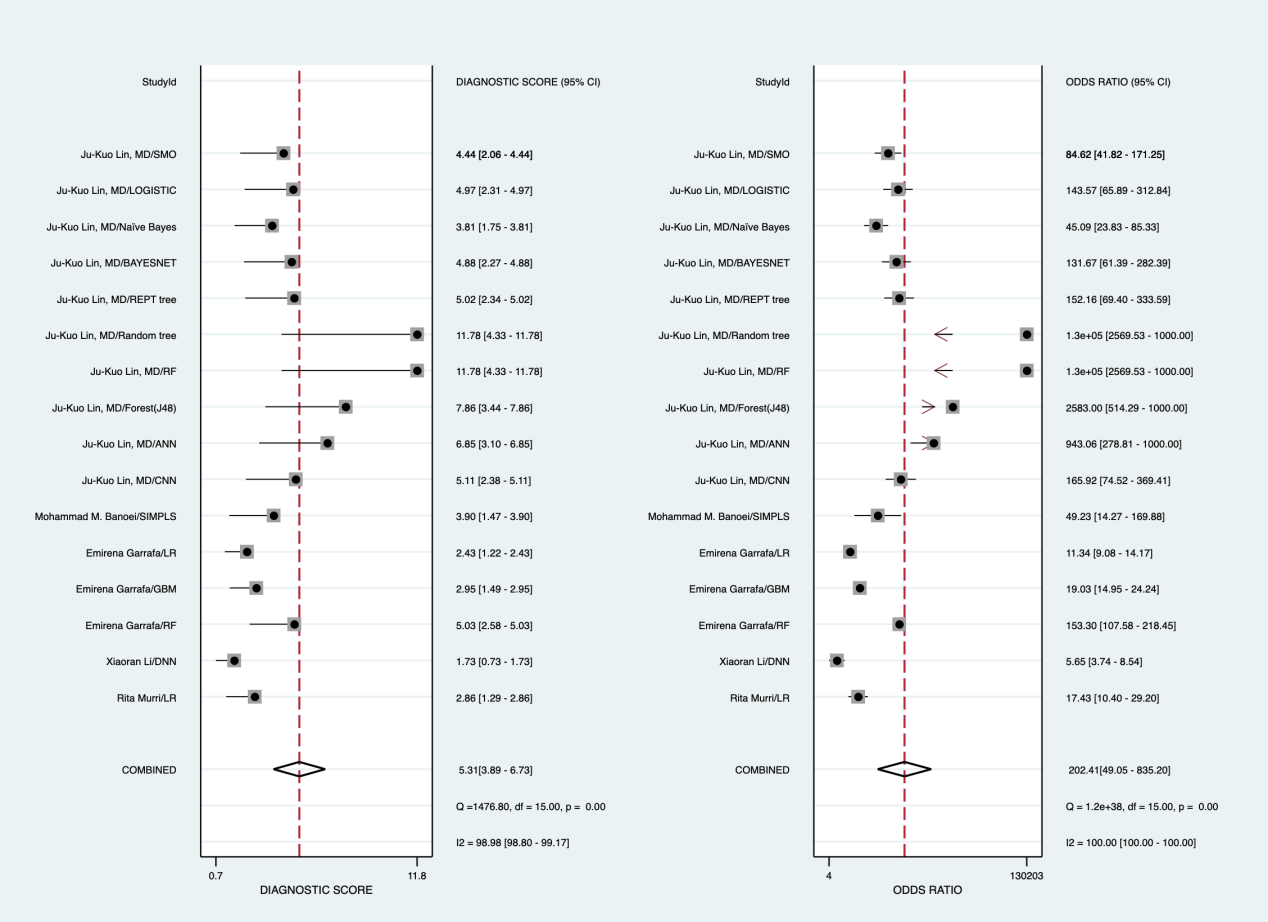


**Figure s8 SROC of AI for the diagnosis of COVID-19 patient mortality for all AI models training sets**


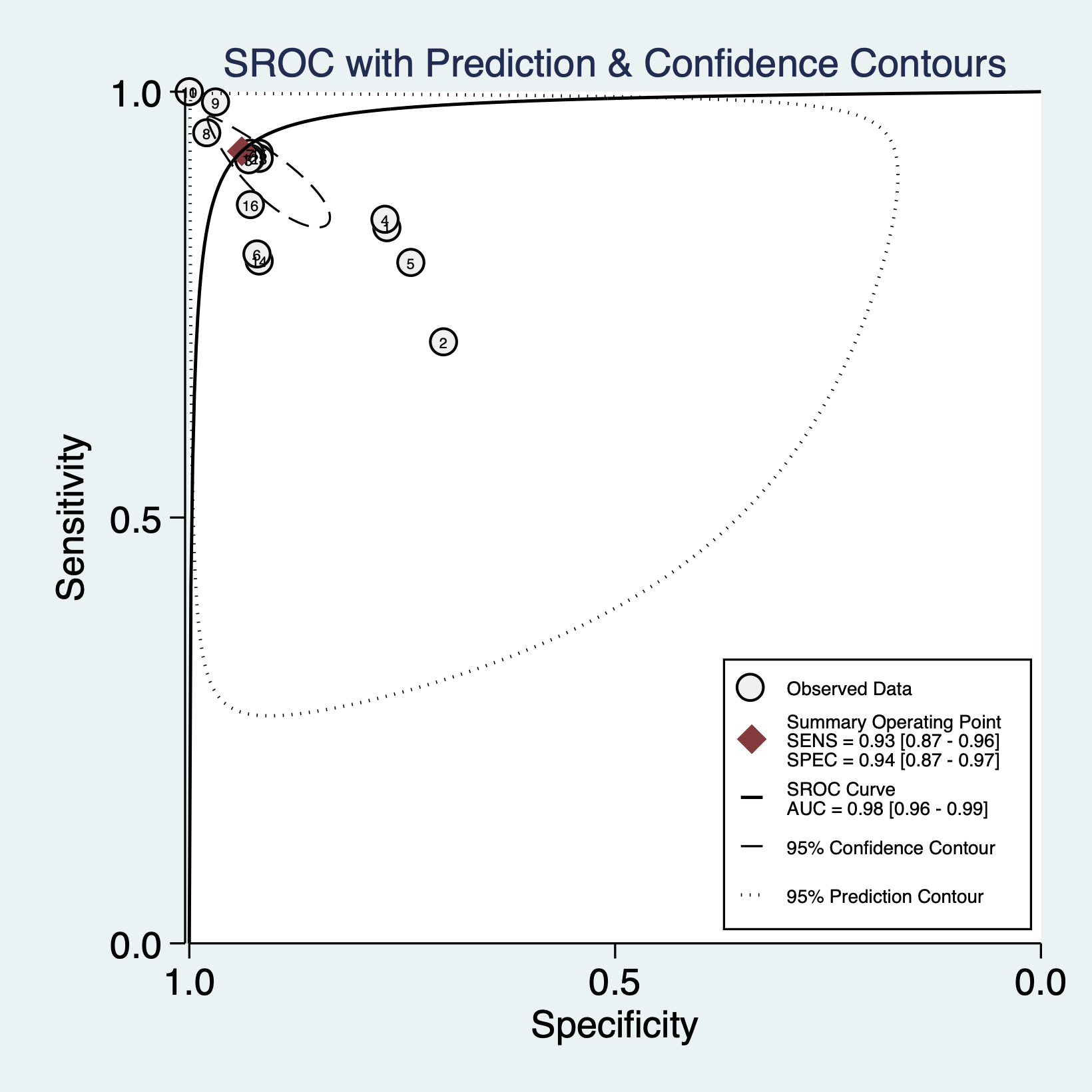


**Figure s9 The analysis of sensitivity**


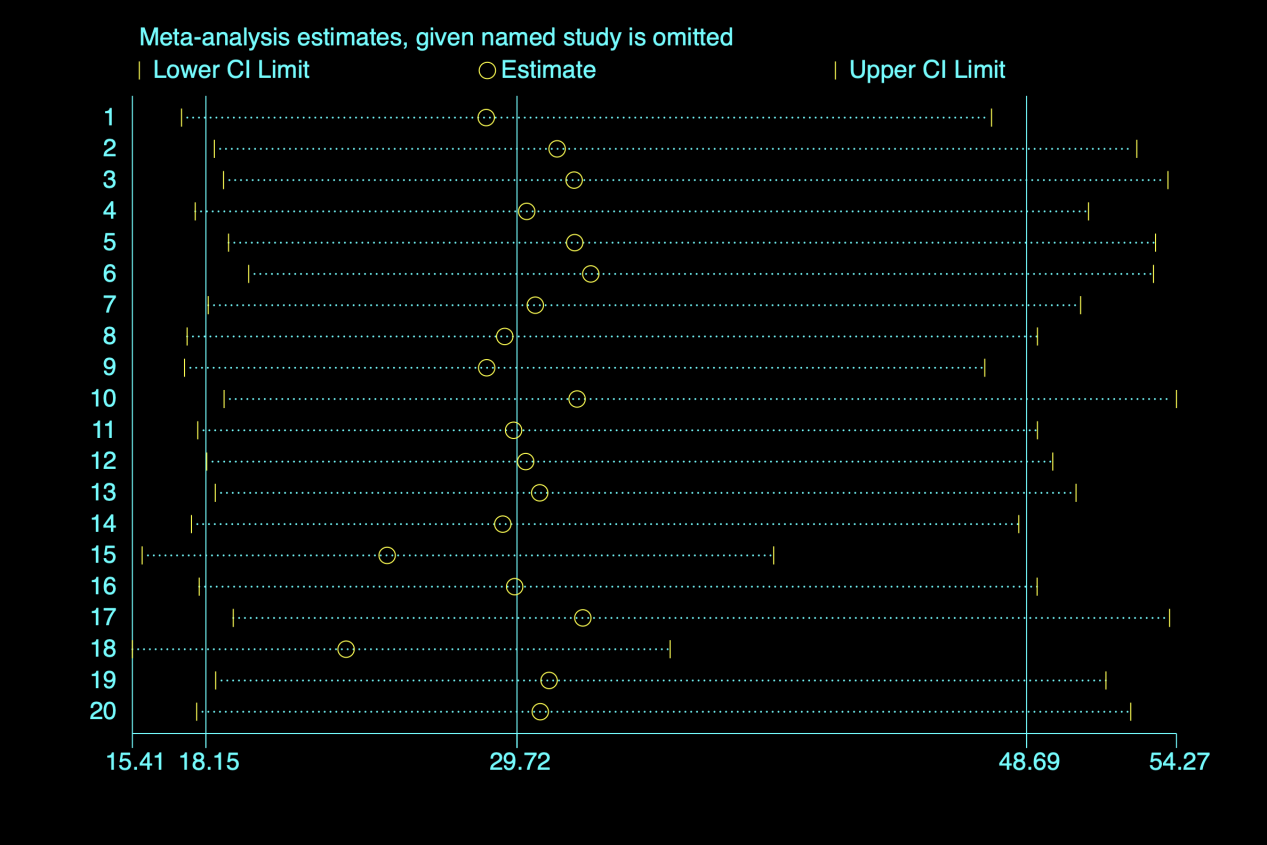


**Table legends:**

Table s1: Validation set basic information

Table s2: Training set basic information

**Figure legends:**

Figure s1: Forest plots of the pooled sensitivity and specificity for all AI models validation sets

Figure s2: Forest plot of the pooled positive LR and negative LR for all AI models validation sets

Figure s3: Forest plot of the pooled diagnostic odds ratio for all AI models validation sets

Figure s4: SROC of AI for the diagnosis of COVID-19 patient mortality for all AI models validation sets

Figure s5: Forest plots of the pooled sensitivity and specificity for all AI models training sets

Figure s6: Forest plot of the pooled positive LR and negative LR for all AI models training sets

Figure s7: Forest plot of the pooled diagnostic odds ratio for all AI models training sets

Figure s8: SROC of AI for the diagnosis of COVID-19 patient mortality for all AI models training sets

Figure s9: The analysis of sensitivity
