# Supplementary material for: The accuracy of artificial intelligence in predicting COVID-19 patient mortality: a systematic review and meta-analysis
Source: BMC Med Inform Decis Mak. 2023 Aug 9;23:155. doi: 10.1186/s12911-023-02256-7 (PMC10410953; doi:10.1186/s12911-023-02256-7)
Supplement: Supplementary file 6 — Supplementary Material 6：Risk of bias assessments [file 12911_2023_2256_MOESM6_ESM.docx]

**Figure S10. Risk of bias and applicability concerns graph**

**
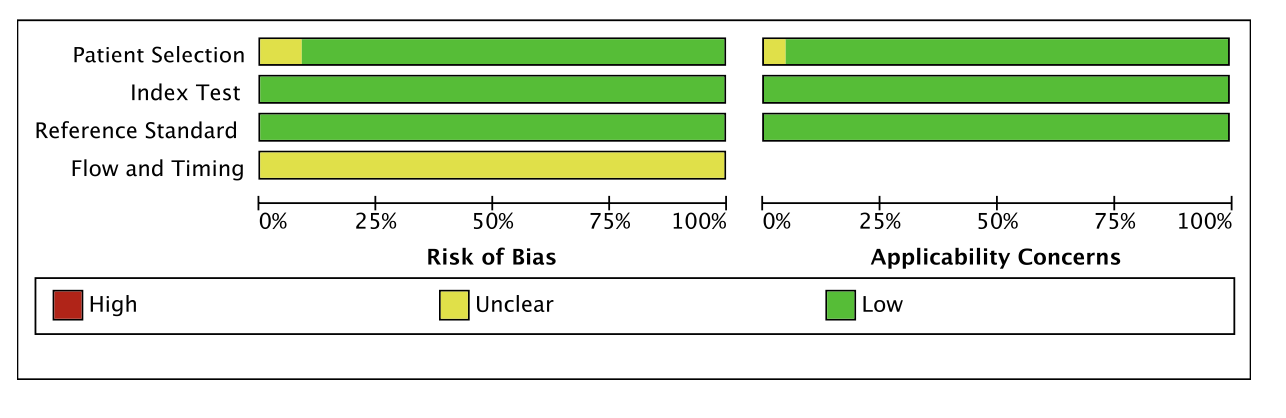
Figure S11. Risk of bias and applicability concerns summary**

**
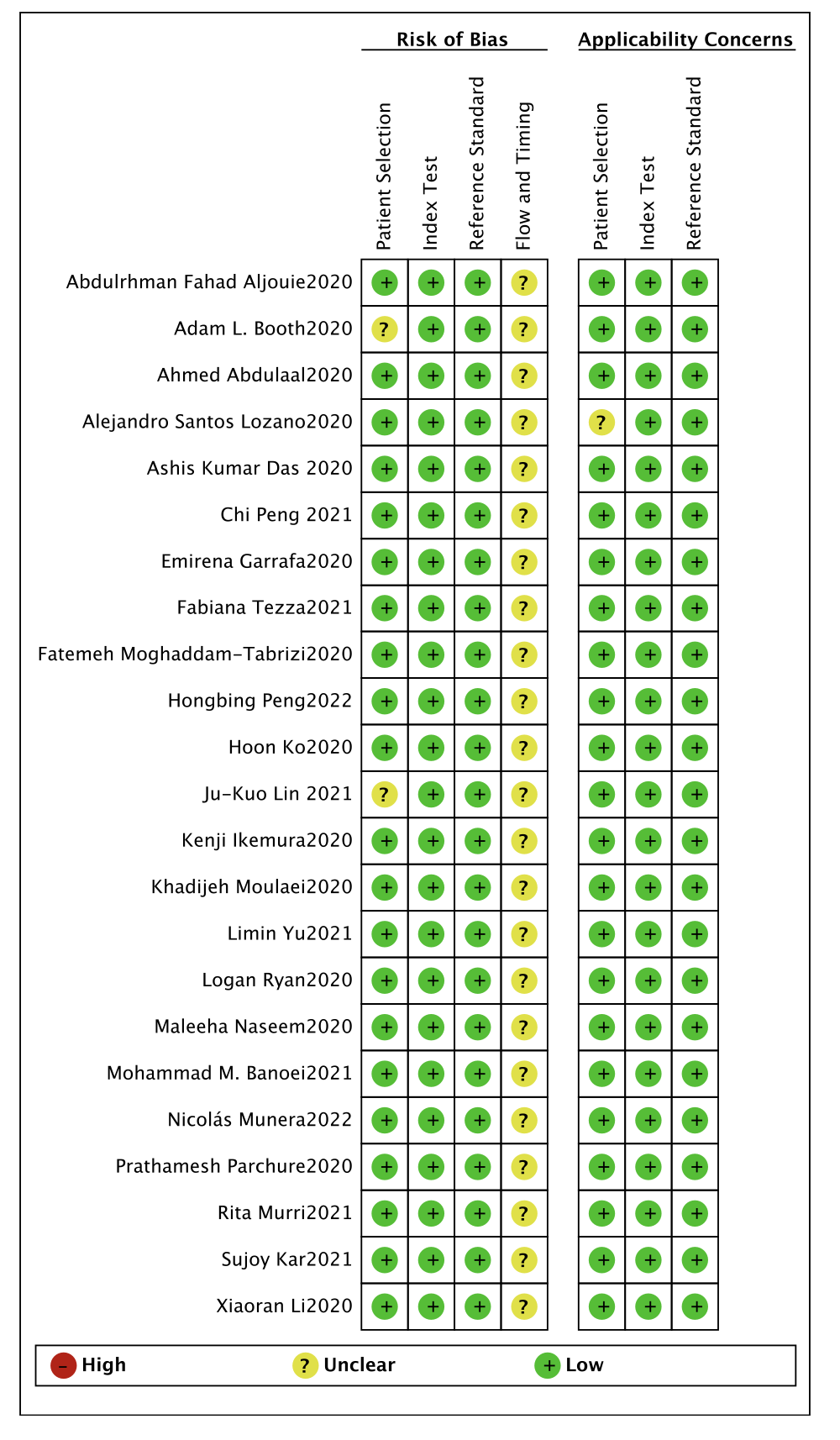
**

**Figure legends:**

Figure S10: Risk of bias and applicability concerns graph

Figure S11: Risk of bias and applicability concerns summary
